# Combined effects of ciprofloxacin and microplastics on alpine spring water microbiota: evidence from glacier-fed microcosm experiments

**DOI:** 10.3389/fmicb.2025.1654589

**Published:** 2025-09-04

**Authors:** Domenica Mosca Angelucci, Federica Piergiacomo, Enrica Donati, Leonardo Pagani, Elisa Minuti, Lorenzo Brusetti, Maria Concetta Tomei

**Affiliations:** ^1^Water Research Institute, National Research Council (CNR-IRSA), Rome, Italy; ^2^Faculty of Agricultural, Environmental and Food Sciences, Free University of Bozen-Bolzano, Bolzano, Italy; ^3^Institute for Biological Systems, National Research Council (CNR-ISB), Rome, Italy; ^4^Infectious Diseases Unit, Central Hospital of Bolzano-Bozen, Bolzano, Italy

**Keywords:** antibiotic resistance, freshwater, microplastics pollution, alpine ecosystem, emerging contaminants, polyethylene terephthalate

## Abstract

**Introduction:**

Emerging contaminants such as microplastics (MPs) and antibiotics pose increasing environmental and public health risks due to their persistence and incomplete removal by wastewater treatment processes. MPs can act as vectors for antibiotics, facilitating their environmental spreading and supporting biofilm formation, which can enhance horizontal gene transfer and antibiotic resistance. This study investigates the combined effects of ciprofloxacin (CIP) and polyethylene terephthalate (PET) MPs on microbiota in alpine spring water (SW) sourced from a rock glacier.

**Methods:**

Four experimental scenarios (Control, CIP, PET, CIP + PET) were established to assess the sorption dynamics of CIP onto PET particles and the consequent microbial responses. A multidisciplinary analytical approach combining ultra-performance liquid chromatography, microscopy, quantitative PCR, and metabarcoding was applied.

**Results:**

CIP exhibited progressive sorption onto PET, accompanied by a time-dependent increase in biofilm formation, most pronounced in the CIP + PET condition. qPCR revealed elevated copy numbers of resistance genes *qnrA* and *qnrB* in CIP + PET, suggesting synergistic effects between antibiotics and MPs in promoting resistance. CIP was the dominant driver of microbial compositional shifts, favoring known CIP-degrading taxa. A shared core microbiome of 216 amplicon sequence variants was detected across all conditions, but specific taxa were differentially enriched under varying exposures. The combined CIP + PET test induced the strongest community shifts, while CIP alone shared fewer taxa with controls, indicating selective pressure for resistant microorganisms like *Achromobacter*. PET MPs also shaped distinct microbial assemblages, possibly by offering niches favoring biofilm-associated genera such as *Luteolibacter*. Biodiversity metrics showed highest richness and evenness in CIP-free conditions (Control and PET), while CIP significantly reduced alpha diversity, favoring resistant taxa, as confirmed by NMDS and lower Shannon and Simpson indices. Effects of MPs were still noticeable.

**Conclusion:**

These findings demonstrate the disruptive effects of CIP on alpine freshwater microbial communities and highlight the additional, though more moderate, influence of MPs. The combined presence of MPs and antibiotics may exacerbate resistance spreading by enhancing persistence and providing favorable conditions for resistant biofilms. A mechanistic understanding of these interactions is essential for accurate risk assessment and the development of effective mitigation strategies in alpine and other vulnerable freshwater ecosystems.

## 1 Introduction

Emerging contaminants such as antibiotics and microplastics (MPs) are increasingly detected across a wide range of environmental matrices and pose growing threats to ecosystem integrity and public health (Niu et al., 2022; Ramírez-Malule et al., 2020; Wang F. et al., 2024). These pollutants originate from diverse anthropogenic sources, including wastewater treatment plants, industrial effluents, aquaculture, and agricultural runoff, and can eventually enter the human food chain (Dessì et al., 2024).

Among these, antibiotics are widely used in human and veterinary medicine and aquaculture, contributing to their pervasive presence in aquatic and terrestrial environments. Ciprofloxacin (CIP), a fluoroquinolone antibiotic, is one of the most frequently detected compounds in environments. Its concentrations range from low ng/L in surface waters to several μg/L in effluent-impacted zones, and up to 0.4 mg/kg in soils (Girardi et al., 2011). While CIP is highly effective against a broad spectrum of bacteria, its misuse and overuse have contributed to the rise of antibiotic-resistant strains, exacerbating the global environmental antimicrobial resistance (AMR) crisis (Dessì et al., 2024; Niu et al., 2022).

CIP resistance mechanisms include chromosomal mutations in DNA gyrase (*gyrA*) and topoisomerase IV (*parC*), overexpression of efflux pumps, and the acquisition of plasmid-mediated quinolone resistance (PMQR) genes such as *qnrA*, *qnrB*, *qnrS*, and *qnrVC*. These genes encode pentapeptide repeat proteins that protect DNA gyrase and topoisomerase IV from quinolone action (Garoff et al., 2018; Shariati et al., 2022). Alarmingly, even low environmental concentrations of CIP (5–10 μg/L) have been shown effective to select for resistant bacteria via chromosomal mutations (Kraupner et al., 2018), highlighting its potential ecological impact.

Microplastics (MPs), defined as plastic particles smaller than 5 mm in size, are persistent environmental pollutants derived from the breakdown of larger plastic debris or released directly as primary MPs. Their high surface-area-to-volume ratio and hydrophobicity facilitate the sorption of various micropollutants, including heavy metals, organic chemicals, pathogens, and antibiotic resistance genes (ARGs) (Mosca Angelucci and Tomei, 2022; Hoang et al., 2024; Rafa et al., 2024). MPs demonstrated sorption capacities several orders of magnitude greater than sediments or water (Sun et al., 2023), enabling them to act as vectors for contaminant transport inside and across environmental compartments.

Beyond their chemical interactions, MPs significantly influence microbial ecology by providing surfaces that support biofilm formation. These biofilm harbor dense and diverse microbial communities capable of enhanced nutrient uptake, survival under environmental stress, and horizontal gene transfer (HGT) (Piergiacomo et al., 2022; Yang et al., 2019). MPs can also carry higher abundances of multidrug resistance genes than surrounding water, thus contributing to the spreading and co-selection of ARGs.

Emerging evidences indicate that MPs can sorb CIP under environmentally relevant conditions, potentially enhancing its persistence and altering its bioavailability (Sun et al., 2023). However, most studies have been conducted in highly controlled laboratory conditions using pristine MPs and ultrapure water and have largely focused on physicochemical mechanisms such as polymer type, pH, and salinity. As such, the biological consequences of CIP-MP co-occurrence on natural microbial communities remain poorly understood.

The combined presence of CIP and MPs raises concerns about their synergistic toxicity and their role in shaping microbial resistance *in situ*. Their interactions are especially relevant in high-mountain environments, where climate change is accelerating glacier retreat and reshaping hydrological regimes. As glaciers or rock glaciers melt, they may release long-time sequestered metal ions, organic compounds or microbial genes, including natural antibiotics, MPs, and ARGs, into freshwater systems, thereby altering downstream microbial assemblages (Segawa et al., 2013; Bach et al., 2018). Indeed, recent studies have confirmed the plastic pollution in remote alpine areas. For example, between 131 and 162 million plastic items were estimated to be in supraglacial debris of the Italian Alps (Ambrosini et al., 2019), while polyethylene terephthalate (PET) MPs were recently detected in snow from the Carnic Alps in northeastern Italy (Pastorino et al., 2021).

Despite growing awareness, the interplay between emerging contaminants, climate-driven environmental change in alpine regions, and the evolution and spread of resistance genes remain largely uncharacterized. Closing this knowledge gap is critical for understanding contaminant pathways, assessing ecological risks, and informing evidence-based mitigation strategies (Buta et al., 2021; Feng et al., 2021).

In this study, we investigated the combined effects of CIP and PET MPs on microbial community dynamics in spring water (SW) collected from a rock glacier in the Italian Alps. In the first part of the study we verified the affinity CIP - PET MPs by applying the Hansen Solubility Parameters (HSP) method. To the best of our knowledge, this is the first study applying the HSP method to pre-select MPs for targeted sorption testing of a specific antibiotic under environmentally relevant conditions. In fact, previous applications of HSP in environmental studies have primarily focused on polymer selection for the removal of organic pollutants from wastewater (Khansary et al., 2017; Pittman et al., 2015; Poleo and Daugulis, 2014; Tomei et al., 2017).

We also hypothesized that the co-presence of CIP and MPs could exerts additive and/or synergistic selective pressures promoting the proliferation of resistant and biofilm-forming taxa while reducing overall bacterial diversity. Moreover, we anticipated that MPs could serve as ecological niches for microbial colonization and HGT, thereby enhancing ARG persistence and dissemination in presence of antibiotics. Using microcosms experiments whose experimental design has been planned according to specialized literature, we evaluated: (i) sorption of CIP onto PET MPs, (ii) synergistic effects on the shifts in bacterial taxonomic composition and potential related bacterial functionalities, (iii) presence and evolution of ARGs.

The results of this work provide novel insights into the fate of relevant emerging contaminants such as antibiotics and MPs and on the ecological risks derived by their co-presence in glacier-influenced aquatic systems. Broader implications for environmental health and AMR in the context of the climate change scenario are also highlighted.

## 2 Materials and methods

### 2.1 Spring water collection and analysis

A total of 14 L of fresh water was collected from the Gadria stream located in the municipality of Lasa (BZ), Italy (coordinates: 46°39′2″N, 10°42′25″E), using sterilized glass bottles. To ensure representative sampling, water was drawn from multiple points along the stream at varying elevations. Upon arrival at the laboratory, all samples were pooled and thoroughly homogenized. The pH of the composite sample was measured in duplicate using a BASIC 20 + pH Meter (Crison), and the average of two readings was 7.59.

### 2.2 Pre-enrichment of freshwater

Due to the low microbial load and minimal sediment content in the SW, microbial communities were pre-enriched by adding a 2.5% yeast extract solution (25 g/L) (Sigma-Aldrich, Merck, United States), following the method described by Bomberg et al. (2020). Specifically, 500 mL of this solution were added to 2 L of stream water, yielding a final yeast extract concentration of 0.5% (5 g/L).

### 2.3 Ciprofloxacin stock solution

CIP powder (Sigma-Aldrich, Merck, USA) was dissolved in pre-enriched freshwater in such amount to obtain a final concentration of 8 mg/L. The solution was mixed overnight with a magnetic stirrer to ensure complete dissolution. This concentration was selected to induce pressure on microbial communities and it is high enough to allow the collection of significant quantitative data in the duration time of the tests. Moreover, the target CIP concentration is within the range (0.1–50 mg/L) of tested concentrations in previous studies dealing with CIP sorption by MPs as reported in Supplementary Table S1.

### 2.4 Microplastics (PET)

PET MPs were prepared by cutting 0.5 L water bottles in squared pieces of ~ 4 × 4 mm. These were washed twice with distilled water and once with 70% ethanol solution, then dried overnight at 55 °C. The dried pieces were stored wrapped in aluminum foil under a biological hood. Plastic fragments of 4 × 4 mm were selected to balance environmental relevance (i.e., dimensions within the microplastic definition <5 mm) with experimental practicality. This size enabled precise handling, reproducible experimental conditions, and robust quantification of pollutant sorption and biofilm development. The use of standardized particles also minimized variability linked to particle size and morphology, thus reducing experimental noise and obviating the need for effect size corrections in data interpretation (Shamskhany et al., 2021).

### 2.5 Microcosm setup

Four scenarios were established in sterile microcosms to assess the individual and combined effects of CIP and PET MPs on freshwater microbial communities:

- Control (A): 250 mL of unamended Rio Gadria SW, without CIP or PET. This setup was replicated three times, and repeated in four experimental runs (total volume: 3.0 L).
- CIP only (B): 100 mL of SW amended with CIP (8 mg/L), without PET. This condition was replicated three times in four runs (total volume: 1.2 L).
- PET only (C): 250 mL of SW with 1 g/L of PET MPs, without CIP. This condition was replicated four times in four runs (total volume: 4.0 L).
- CIP + PET (D): 250 mL of SW with both CIP (8 mg/L) and PET (1 g/L). This condition was replicated four times in four runs (total volume: 4.0 L).

Tests with PET have been performed at 1 g/L concentration to have enough plastic to perform all the required tests in the small volume of the microcosms. This condition has been chosen in accordance to the reported range (i.e., 0.1–100 g/L) of tested concentrations in MP-CIP previous studies shown in Supplementary Table S1, and allowed achieving quantitative results high enough to investigate all the involved chemical and microbiological processes, and to perform comparative analyses with previous studies.

Microcosms were sealed with sterile gauze and hydrophilic cotton stoppers, which prevented contamination while maintaining aerobic conditions. All microcosms were designed to simulate environmentally relevant conditions typical of temperate freshwater and lentic systems, characterized by moderate temperature (~20 °C), absence of light, and static conditions for 60 days (Schiraldi and De Rosa (2014), Maberly et al., 2020, Kim et al., 2020, Roy et al., 2021; Ylla et al., 2009; Merritt et al., 2005; Han and Lee, 2023). Similar incubation parameters have also been adopted in previous microcosm studies investigating microbial colonization and pollutant dynamics on microplastics (e.g., Zadjelovic et al., 2023). Samples were periodically agitated to maintain homogeneity.

#### 2.5.1 Microcosm sampling

Sampling was performed on days 10, 20, 40, and 60. For microcosms without PET, 12 mL of water was filtered through a 0.2 μm syringe filter and stored in 15 mL Falcon tubes for subsequent CIP concentration analysis. The remainder was filtered using a 47 mm, 0.2 μm membrane (Whatman), and the filters were stored for DNA extraction. Excess water was frozen at −80 °C for potential further analysis. For microcosms with PET, water samples for CIP analysis were processed similarly. PET pieces were stored in 2 mL tubes for CIP content quantification, and five pieces were fixed in 4% paraformaldehyde (1: 1 ethanol: PBS) and stored at −20 °C for microscopy. The remainder was filtered and processed as above, and any biofilm on the PET was collected using a sterile loop and added to the DNA extraction tube. Excess water was stored at −20 °C.

### 2.6 Chemical and microbiological analysis

#### 2.6.1 CIP quantification in aqueous samples

Chromatographic analyses were conducted using an Acquity™ UPLC H-Class Bio system (Waters, Milford, United States), equipped with a BEH C18 column (50 × 2.1 mm, 1.7 μm) and a matching pre-column. Elution was performed using a gradient of 0.1% formic acid in water (phase A) and acetonitrile (phase B): 0–0.5 min (20% B), 0.5–1 min (90% B), 2 min (90% B), followed by re-equilibration to 20% B. The flow rate was 0.3 mL/min, the sample injection volume was 2 μL and detection was 278 nm. The optimized method was validated in terms of linearity, sensitivity, and precision. CIP stock standard solution was prepared in water containing 1% formic acid. Calibration was linear between 0.045 and 30 μg/mL (*n* = 5; *R*^2^ = 0.9994), with a limit of detection of 0.015 μg/mL and a limit of quantitation of 0.045 μg/mL. Precision (relative standard deviation) was <1% intraday and <2% interday, with six consecutive injections.

#### 2.6.2 CIP quantification in polymeric samples

PET samples were weighed and added to a 2 mL polypropylene (PP) centrifuge tube, then extracted in 1 mL of a 1% (v/v) formic acid in methanol. Extracted samples were vortexed, ultrasonicated for 10 min, and centrifuged at 13,000 rpm for 5 min. This process was repeated three times, and the supernatants were pooled, evaporated under nitrogen, and redissolved in MeOH: H_2_O (50:50, 1% formic acid). The extract was filtered (0.2 μm nylon membrane; Sartorius, Göttingen, Germany) before UPLC analysis.

#### 2.6.3 DNA extraction and quantification

DNA was extracted from membrane filters and biofilms using the DNeasy^®^ PowerSoil^®^ Pro Kit (QIAGEN, Milan, Italy), following the manufacturer’s protocol. DNA was quantified using a Qubit^®^ 2.0 Fluorometer (Invitrogen, Thermo Fisher Scientific, Monza, Italy) and verified by 1.5% agarose gel electrophoresis. DNA fragment integrity was visualized with the GelDoc Go Imaging System (BioRad, Segrate, Italy).

#### 2.6.4 Microscopy of biofilms on PET

Biofilms on PET fragments were assessed via DAPI (Sigma-Aldrich, Merck, United States) staining and epifluorescence microscopy. After fixation, samples were washed twice with PBS, stained with 10 μL of DAPI (10 μg/mL), and incubated in the dark at 4 °C for 20 min. Stained PET fragments were mounted on slides using fungal tape. Imaging was performed with a LEICA DM2500 LED microscope (LEICA Microsystems, Buccinasco, Italy) (359 nm excitation, 461 nm emission), and captured with a LEICA DFC300 FX camera. Images were processed with LEICA IM50 software.

#### 2.6.5 Quantification of resistance genes via qPCR

Quantitative PCR (qPCR) was used to quantify 16S rRNA and quinolone resistance (*qnrA*, *qnrB*, *qnrC*, *qnrD*, *qnrS*) genes from DNA diluted 1:10. For 16S rRNA gene, reactions used primers 241F and 518R with SYBR Green Master Mix (Applied Biosystems, Monza, Italy), and thermal cycling protocol followed by Muyzer et al. (1993). *qnr* genes were amplified using primers from Rutgersson et al. (2014) and Yengui et al. (2022). Standard curves from 10^8^ to 10^0^ gene copies were prepared using recombinant plasmids (Eurofins MWG Operon, Vimodrone, Italy). All qPCRs were run on a Bio-Rad CFX96™ with technical triplicates and appropriate controls.

#### 2.6.6 16S rRNA gene metabarcoding

Microbial community composition was assessed by Illumina MiSeq sequencing (Biofab s.r.l., Rome, Italy) of the 16S rRNA gene. Sequencing data were processed in R and taxonomically annotated via BLAST. Data were deposited in the NCBI SRA under BioProject accession number PRJNA1241897.

### 2.7 Hansen solubility parameters method

Available data from specialized literature (Supplementary Table S1) were insufficient to unequivocally identify the optimal MP for this study in terms of CIP sorption capacity. Therefore, we adopted a theoretical approach using HSPs to predict the thermodynamic affinity between polymers and organic compounds, as suggested by Mosca Angelucci and Tomei (2022). These parameters are typically tabulated for commercial polymers (Hansen, 2007) and environmentally relevant compounds, such as CIP (Silva et al., 2018). Solubility parameters quantify the interactions through the sum of dispersive (*δ*_D_), polar (δ_P_), and hydrogen-bonding (δ_H_) forces. From the solubility parameters, the interaction radius (Ra), is calculated as Equation 1:


(1) $$\mathrm{Ra}=\sqrt{\left[4\cdot {\left(\delta_{D,P}-\delta_{D,C}\right)}^{2}+{\left(\delta_{P,P}-\delta_{P,C}\right)}^{2}+{\left(\delta_{H,P}-\delta_{H,C}\right)}^{2}\right]}$$

where subscripts P and C indicate polymer and compound, respectively. Lower Ra values indicate higher thermodynamic affinity.

### 2.8 Statistical analysis

All statistical analyses were conducted using PAST (version 3.0; Hammer et al., 2001) and R (version 4.4.2; R Core Team, 2024). Multivariate analyses of beta diversity, including PERMANOVA (*adonis*) and tests for homogeneity of group dispersion (*betadisper*), were performed using the vegan package. Differences in alpha diversity metrics and taxonomic abundances among treatments were assessed using non-parametric Kruskal–Wallis tests, followed by Dunn’s *post hoc* comparisons with Bonferroni correction, implemented through the FSA and rstatix packages. Indicator Species Analysis was carried out with the indicspecies package using the multipatt() function, with 999 permutations to evaluate significance. Results were considered statistically significant at *p* < 0.05 unless otherwise stated.

## 3 Results and discussion

### 3.1 PET-CIP interactions and biofilm dynamics

#### 3.1.1 Affinity CIP-PET

**Figure 1:**
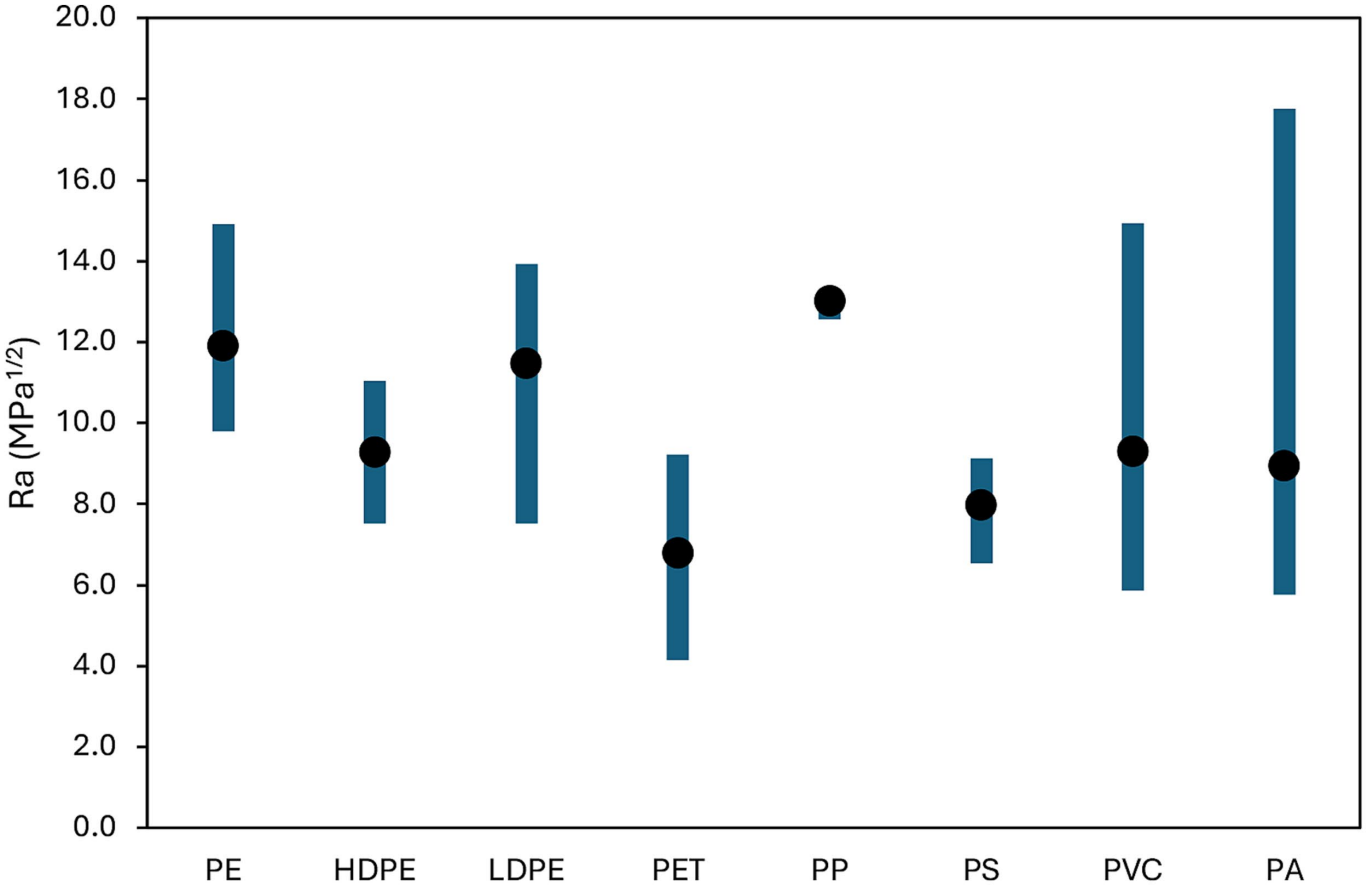
Minimum, maximum and average Ra values estimated for each MP-CIP pair.

The Hansen method based on solubility parameters identified PET as the polymer with the highest affinity for CIP. Ra values for several polymers, i.e., high-density polyethylene (HDPE), low-density polyethylene (LDPE), polyamide (PA), polyethylene (PE), PET, PP, polystyrene (PS) and polyvinyl chloride (PVC) are summarized in Supplementary Table S2, while each combination is reported in Figure 1, yielding the affinity ranking: PP < PE < LDPE < HDPE = PVC < PA < PS < PET.

CIP is a polar compound containing several functional groups like carboxylic and ketone groups, a piperazine ring and a fluorinated aromatic ring contributing to strong polar and hydrogen bonding capabilities. Among tested polymers, PET exhibits polarity (δ_P_) though ester linkages and hydrogen bonding capability (δ_H_) with carbonyl and ester groups, besides an aromatic character from the benzene ring in its backbone. These structural characteristics align well with CIP features of: (i) hydrogen bonding potential, (ii) polar nature (especially in aqueous solutions) and (iii) aromatic structure enabling *π*–π interactions (Lin et al., 2020). Other tested polymers display lower affinity with CIP than PET because highly nonpolar and with mostly dispersive interactions (e.g., HDPE, LDPE, PE, and PP), slightly polar but low hydrogen bonding (PVC and PS) and, even if characterized by strong hydrogen bonding, PA seems to be too polar with mismatched parameters leading to a higher Ra than PET.

The predicted affinity ranking aligns with empirical findings: Lin et al. (2020) reported that PET had the highest CIP sorption among polymers tested. Enyoh et al. (2023) demonstrated superior sorption for pristine, acid-modified, and aged PET; and Wang W. M. et al. (2024) confirmed PET as a dominant polymer accumulating CIP in aquatic environments.

#### 3.1.2 Biofilm development on PET and in CIP tests

Biofilm formation was early observed at day 10 and persisted throughout the 60-day experiment (Supplementary Figure S1), particularly in CIP-only (B) and CIP + PET (D). In test B, biofilms were suspended in water phase; while in D, they adhered to PET surfaces. Biofilms resisted detachment even with sterilized loops, indicating strong adhesion. Epifluorescence microscopy confirmed dense bacterial communities embedded in extracellular polymeric substance (EPS), with PET providing a stable support for colonization. Epifluorescence images showed increasing biomass over time, especially under CIP pressure, supporting the hypothesis that antibiotics select for biofilm-resilient microbial assemblages (Flemming et al., 2016).

#### 3.1.3 Fate and sorption of CIP

**Figure 2:**
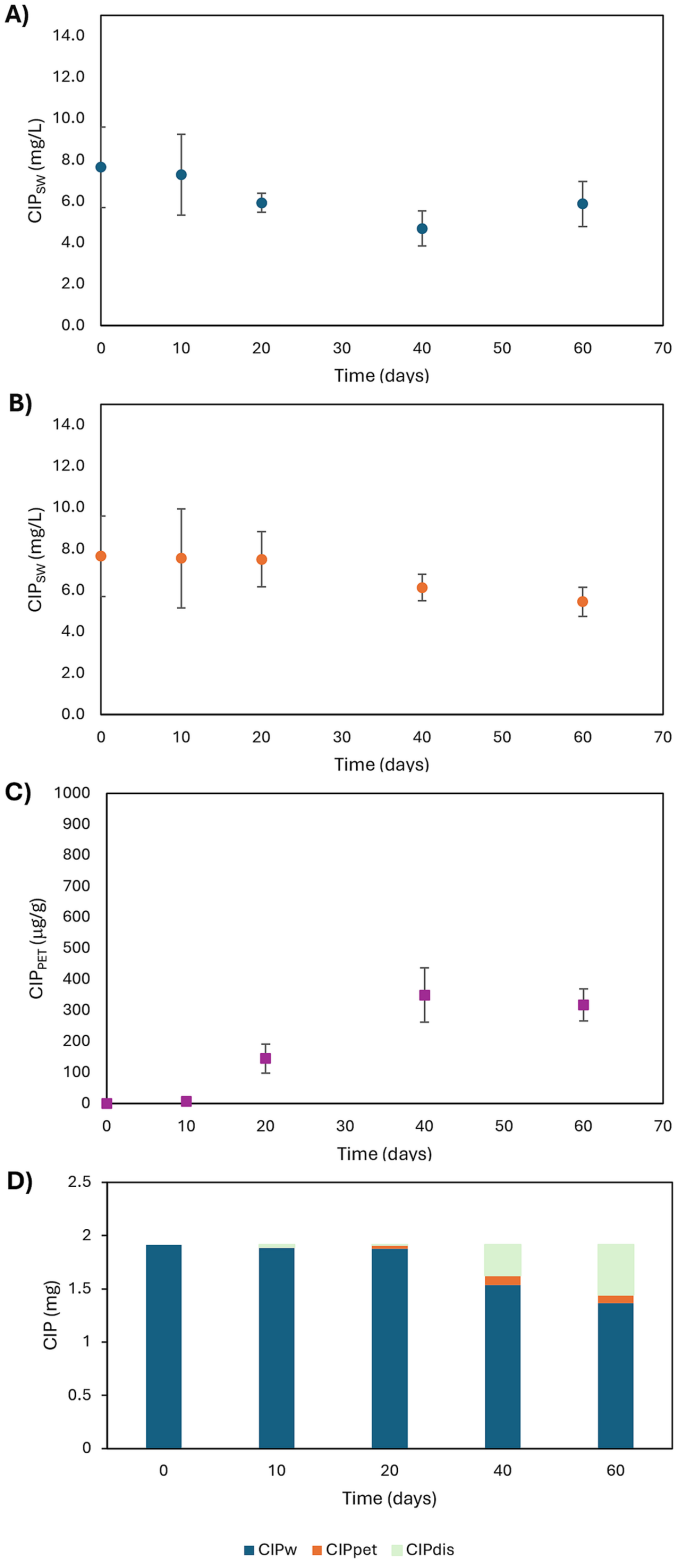
Time profiles of CIP concentrations in aqueous phases of B **(A)** and D **(B)** conditions and in PET MPs **(C)** in D conditions and CIP mass balance in D conditions **(D)**.

CIP concentrations were monitored in both aqueous and solid phases in treatments B and D (Figures 2A–C). PET sorption of CIP was confirmed, with equilibrium reached around day 40 (Figure 2C). CIP concentration in the aqueous phase declined more slowly in D than in B (Figures 2A,B), suggesting PET delayed CIP disappearance. In D, the decrease began only after 20 days, indicating a lag phase in sorption. By day 60, CIP levels in the aqueous phase were lower in D than in B, which may be explained with CIP uptake by PET.

Percent of CIP sorbed onto PET, evaluated with reference to the initial amount of CIP in the aqueous phase, were 0.1, 1.9, 4.5, and 4.2% at 10, 20, 40, and 60 days, respectively. The CIP mass balance was calculated as Equation 2:


(2) $${\left[\mathrm{CI}P_{\mathrm{dis}}\right]}_{t}={\left[\mathrm{CI}P_{\mathrm{SW}}\right]}_{t}\cdot V_{\mathrm{SW}}+{\left[\mathrm{CI}P_{\mathrm{PET}}\right]}_{t}\cdot w_{\mathrm{PET}}$$

where [*CIP_dis_*]*_t_* is the amount of CIP disappeared at time *t*, [*CIP_SW_*]*_t_* and [*CIP_PET_*]*_t_* are the CIP concentrations in liquid and polymeric phases at time *t*, respectively, and *V_SW_* and *w_PET_* are the liquid volume and the PET mass within the microcosm, respectively.

Negligible sorption occurred during the first 10 days, followed by a significant increase peaking at 25% disappearance by day 60 (Figure 2D). This removal likely reflects both competitive sorption or biosorption, and/or biofilm-mediated biodegradation. PET colonization by biofilms may promote stronger interactions with CIP, through an augmented surface area or functional groups facilitating stronger interactions with the antibiotic. He et al. (2023) reported a 50.6% increase in norfloxacin sorption due to biofilm formation on MPs (PVC, PA, and HDPE). Initial low sorption may reflect insufficient biofilm; which, once established, can enhance CIP retention via physical entrapment and metabolic transformation. Zhuang and Wang (2023) highlighted that antibiotic sorption by MPs can be mediated not only by the polymer surfaces themselves, but also, crucially, by the associated biofilms. These biofilms contribute through multiple physicochemical interactions, including hydrophobic forces, van der Waals interactions, electrostatic attractions, *π*–π stacking, and hydrogen bonding. Furthermore, biofilm communities on MPs may promote antibiotic biodegradation via the production of EPS and the acquisition of ARGs, enabling complex biochemical transformation pathways.

Although no CIP degradation products were detected by PDA chromatograms, their absence could result from very low concentrations, sorption into EPS, or complete metabolic turnover. Sorption dynamics on EPS and MPs likely modulate local antibiotic concentrations at the biofilm-plastic interface, potentially influencing the selection of ARGs as discussed in Section 3.3.

### 3.2 Microbial community shifts

#### 3.2.1 Taxonomic composition

It is important to note that biofilm was recovered by scraping the plastic surface with a sterile loop, a method that, while efficient and commonly used in similar studies (Niboucha et al., 2022), may not detach all embedded or strongly adherent microorganisms. Nevertheless, this approach ensures reproducibility, reduces contamination risk, and captures the dominant surface-associated microbial fraction, which plays a central role in interactions with the plastic surface and sorbed pollutants. The observed microbial shifts should therefore be interpreted in the context of this methodological limitation.

**Figure 3:**
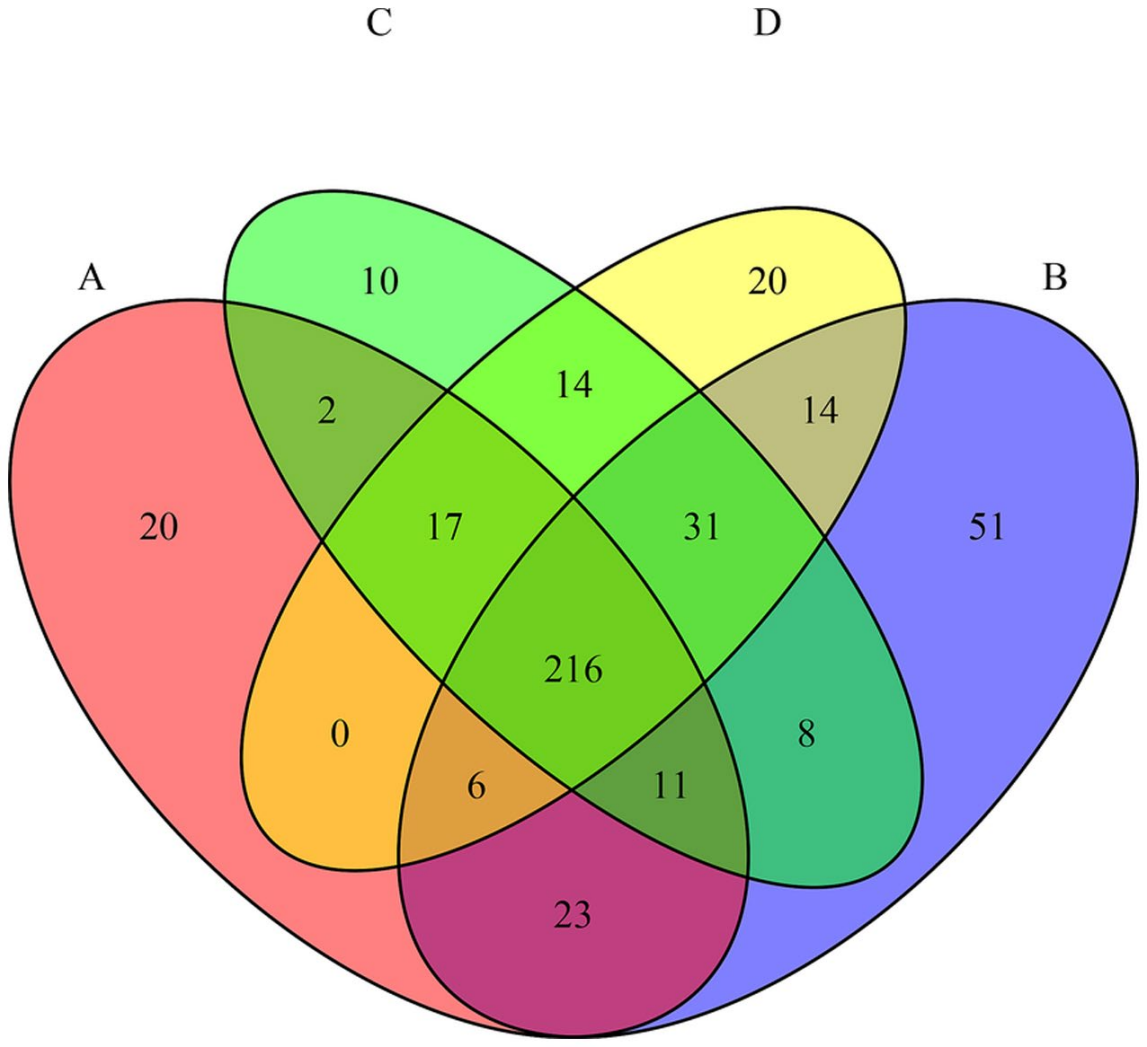
Venn diagram showing the number of shared and unique amplicon sequence variants (ASVs) among the four experimental conditions (A = CTRL; B = CIP; C = PET; D = CIP + PET). The overlaps represent ASVs detected in more than one condition, while non-overlapping areas indicate condition-specific taxa.

Metabarcoding analysis yielded 6,051 ASVs, with an average of 50,440 reads per sample (range: 21,115–93,563). Samples from day 10 were excluded due to insufficient sequencing depth. A core microbiome of 216 taxa was identified across all tests (Figure 3), indicative of a resilient microbial backbone adapted to alpine spring environments and potentially tolerant to environmental stressors (Szekeres et al., 2023; Frey et al., 2016; Peter et al., 2011). Test B (CIP only) harbored the highest number of unique ASVs (*n* = 51), followed by test D (CIP + PET, *n* = 20), reflecting selective pressure by CIP, that suppresses susceptible populations while enriching resistant ones (Sanz-García et al., 2022; Nesse et al., 2023; Campey et al., 2024). The overlap of genera between B and D (*n* = 14) reinforces CIP as the main driver of community shifts. PET also influenced composition: 14 shared genera between C and D imply additive or synergistic effects of combined contaminants (Li et al., 2025; Li et al., 2023; Yu et al., 2022).

**Figure 4:**
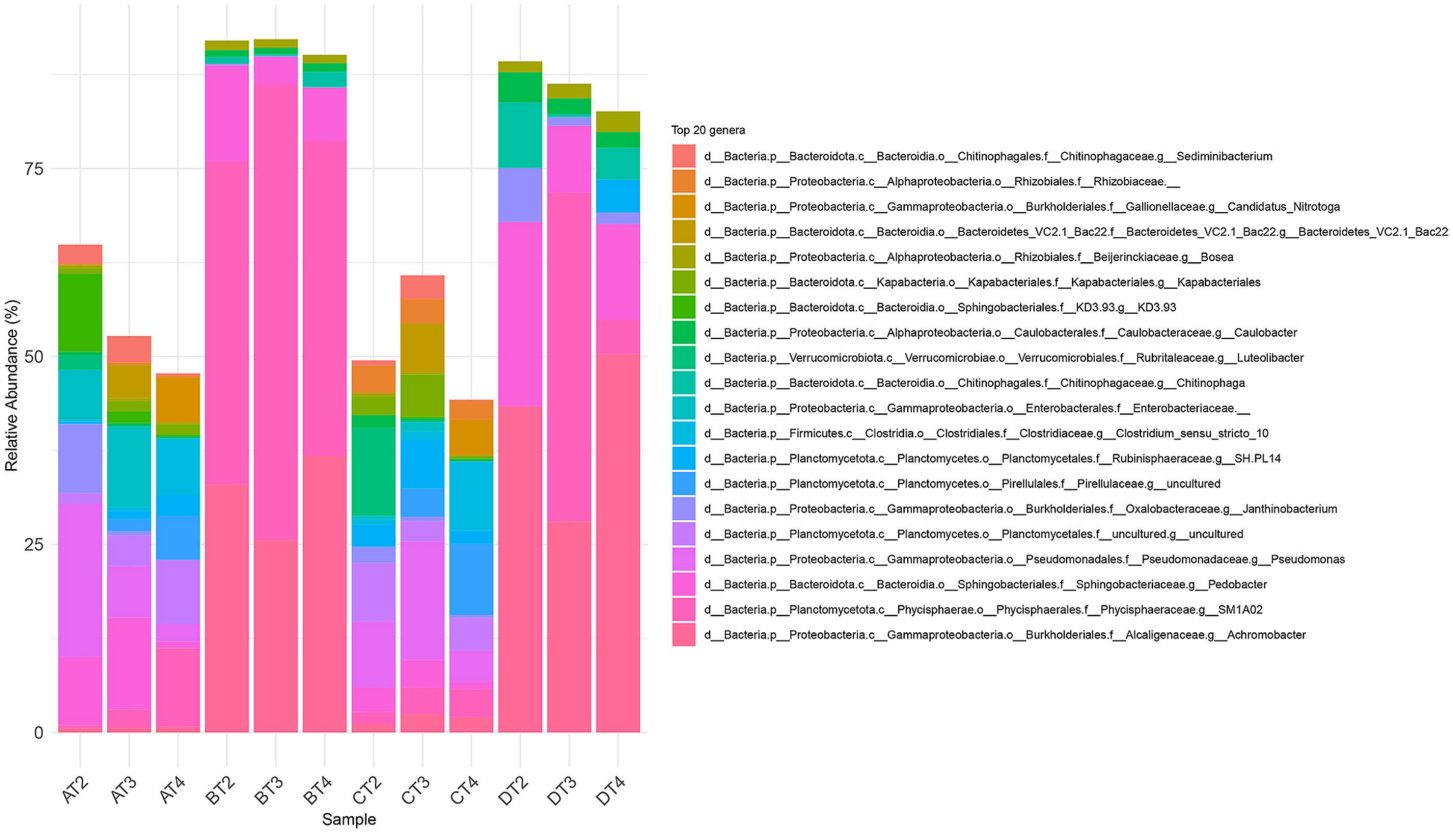
Taxonomic composition of microbial communities at the genus level (20 most prevalent genera) across the four experimental treatments (A: CTRL, B: CIP, C: PET, D: CIP + PET) and four incubation time point (T1: 10 days; T2: 20 days; T3: 40 days; T4: 60 days).

**Figure 5:**
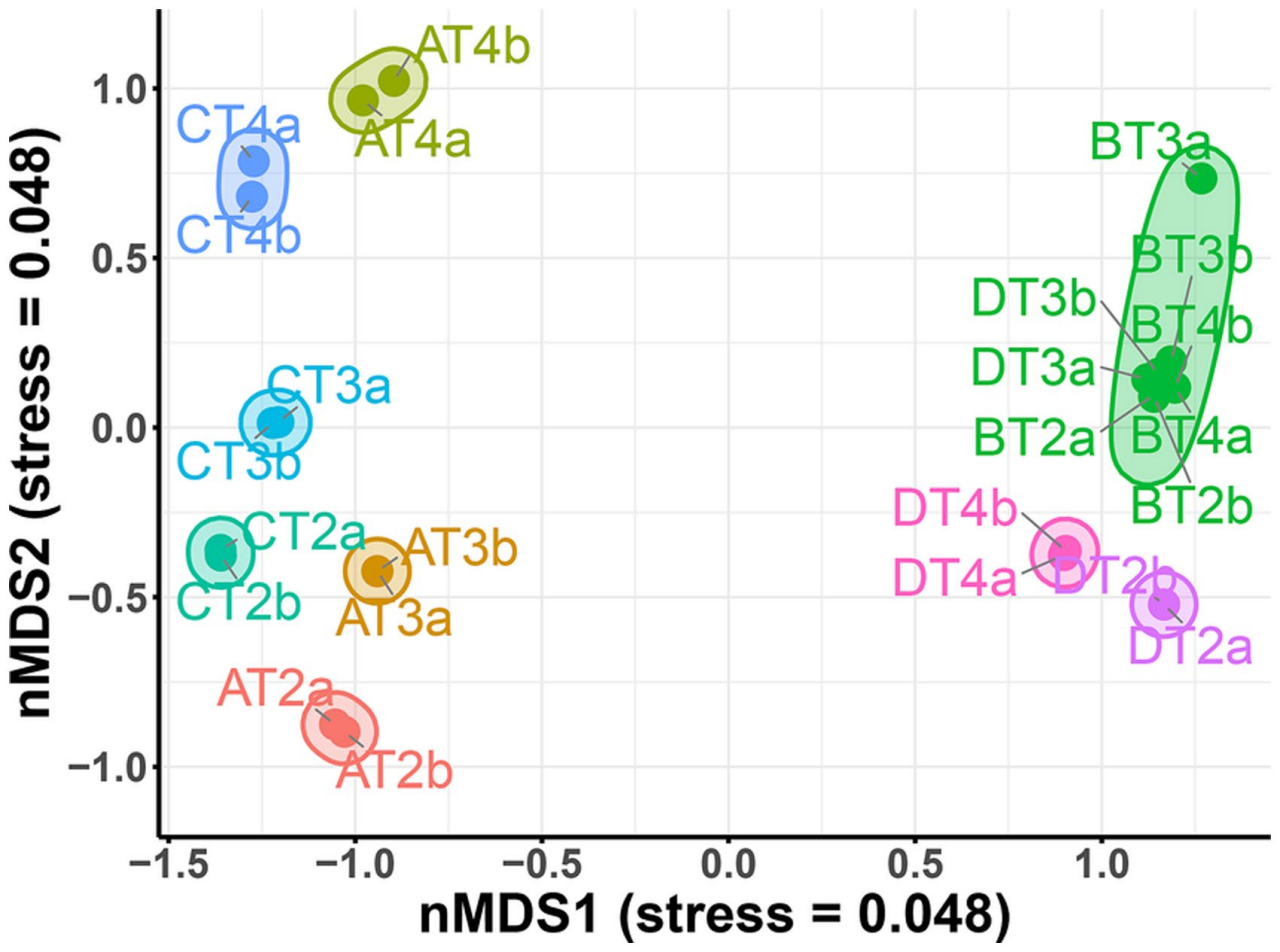
Non-metric multidimensional scaling (NMDS) plot based on Bray–Curtis dissimilarity, illustrating differences in microbial community composition across treatments (A: CTRL, B: CIP, C: PET, D: CIP + PET), timepoints (T1: 10 days; T2: 20 days; T3: 40 days; T4: 60 days) and replicates (a, b). Each point represents a microbial community from a sample, with colors indicating treatment groups. Clustering of samples reflects compositional similarity, with tighter groupings indicating higher similarity in community structure.

The stacked barplot of the 20 most abundant genera (Figure 4) and NMDS ordination (Figure 5) confirmed these shifts. Tests B and D were dominated by *Pedobacter*, *Achromobacter*, and *Physcisphaeraceae*, with lower diversity than control (A), while PET-only (C) and control (A) samples supported more even, taxonomically rich communities, including increased *Rhizobiaceae*, *Luteolibacter*, *Pirullelaceae*, and *Pseudomonas*. *Janthinobacterium* and *Chitinophaga* were enriched in D, indicating CIP’s role in selecting for biofilm-forming, resilient taxa (Madhavan et al., 2024; Trancassini et al., 2014; Wiegand et al., 2020). PET alone also selected for biofilm-associated taxa such as *Rubinisphaeraceae* and *Sediminibacterium*, likely due to the increased surface area facilitating colonization and nutrient adsorption (Yu et al., 2022; Kang et al., 2014). To evaluate the effect of the thesis on microbial community composition, we performed a PERMANOVA based on Bray–Curtis dissimilarities (999 permutations). The analysis revealed a significant effect of the additions (*F* = 17.39, *R*^2^ = 0.723, *p* = 0.001), indicating that they explained approximately 72% of the variation in beta diversity.

To verify the assumption of homogeneity of group dispersions, we conducted a test using betadisper. The ANOVA on multivariate dispersions showed no significant differences among treatments (*F* = 1.08, *p* = 0.381), and this result was confirmed by a permutation test (*F* = 1.08, *p* = 0.364, 999 permutations). These findings support that differences detected by PERMANOVA are not driven by unequal variances among groups (Supplementary Table S3). Indicator Species Analysis was employed to further support these findings, identifying several ASVs significantly associated with specific treatments (*p* < 0.05; Supplementary Table S4). Notably, Chitinophaga was an indicator for CIP-only treatment (B, *p* = 0.008), while *Sediminibacterium* (*p* = 0.008), *Kapabacteriales* (*p* = 0.008), and *Clostridium* sensu stricto 10 (*p* = 0.008) were consistently associated with the control condition (A) and PET-only (C), suggesting a preference for low-antibiotic, high-surface environments. Luteolibacter emerged as a shared indicator across multiple treatments, including PET and CIP (*p* = 0.014), highlighting its adaptability. All identified indicators exhibited high association strength (stat > 0.98), reinforcing the robustness of the treatment-specific microbial responses.

Alpha diversity indices (Table 1) supported these patterns: control (A) maintained high richness and diversity (Taxa: 174 → 288; Shannon: 3.28 → 3.94; Simpson: 0.92 → 0.96; Evenness: 0.15 → 0.18), CIP (B) showed a general diversity reduction (Taxa: 66 → 51; Shannon: 1.55 → 1.57; Simpson: 0.69 → 0.55; Evenness: 0.07 → 0.09), PET (C) preserved diversity comparable to control (Taxa: 227 → 280; Shannon: 3.76 → 4.04; Simpson: 0.96; Evenness: 0.19 → 0.20), and CIP + PET (D) exhibited intermediate diversity (Taxa: 56 → 70; Shannon: 1.77 → 2.03; Simpson: 0.74 → 0.72; Evenness: 0.10 → 0.13), suggesting that PET does not neutralize CIP’s effects directly, but instead facilitates the formation of protective biofilms, which may shield microbial communities from antibiotic exposure.

**Table 1:** Diversity indices (Taxa, Dominance, Simpson, Shannon, Evenness) for the different treatments (A: CTRL, B: CIP, C: PET, D: CIP + PET) and time points (T1: 10 days; T2: 20 days; T3: 40 days; T4: 60 days).

		Taxa	Dominance	Simpson	Shannon	Evenness
	A	174	0.08	0.92	3.28	0.15
	B	66	0.31	0.69	1.55	0.07
T2	C	227	0.04	0.96	3.76	0.19
	D	56	0.26	0.74	1.77	0.10
	A	193	0.04	0.96	3.80	0.23
	B	62	0.45	0.55	1.26	0.06
T3	C	246	0.05	0.95	3.80	0.18
	D	70	0.28	0.72	1.76	0.08
	A	288	0.04	0.96	3.94	0.18
	B	51	0.32	0.68	1.57	0.09
T4	C	280	0.04	0.96	4.04	0.20
	D	57	0.28	0.72	2.03	0.13

To statistically assess differences in alpha diversity across treatments, we applied Kruskal–Wallis tests followed by Dunn’s *post hoc* comparisons (Supplementary Table S5). Significant differences were detected in the Simpson index (*p* = 0.05) and Evenness (*p* = 0.030) between the control (A) and ciprofloxacin-treated sample (B) at time point 3, indicating a reduction in community diversity under antibiotic exposure. Moreover, a significant difference in the number of observed taxa was found at time point 4 (*p* = 0.049), further supporting the impact of ciprofloxacin and the slight effect of microplastics on microbial community structure.

#### 3.2.2 Potential functionalities of bacterial communities

**Figure 6:**
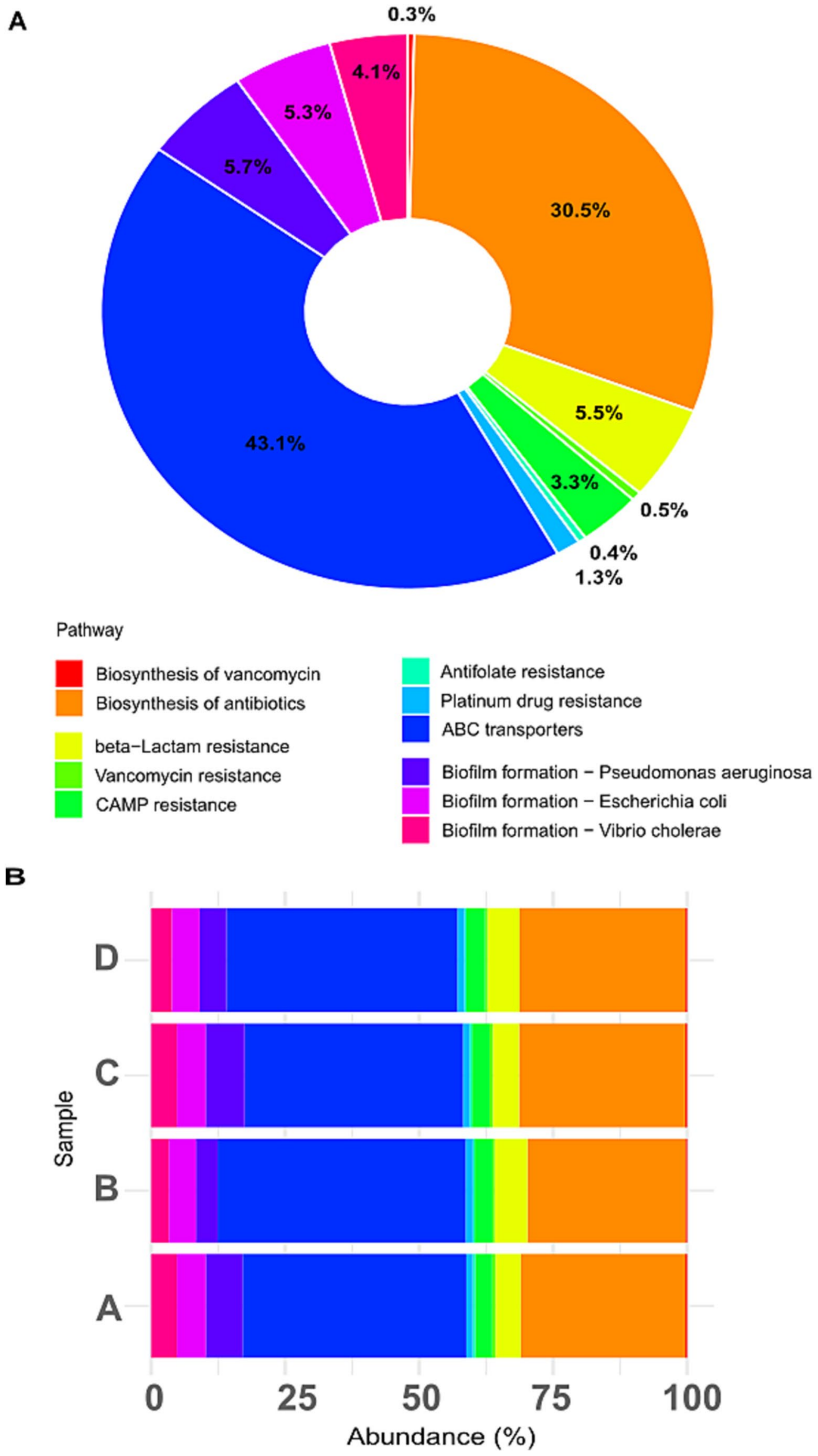
Predicted functional pathways related to antibiotic resistance, biofilm formation, and transporters. **(A)** Donut chart showing the relative abundance of selected KEGG pathways predicted across all samples based on 16S rRNA gene data using the *Tax4Fun* pipeline. Functional categories include antibiotic biosynthesis, various antibiotic resistance mechanisms (e.g., β-lactam, vancomycin, CAMP), biofilm formation in specific bacteria (e.g., *Pseudomonas aeruginosa*, *Escherichia coli*, *Vibrio cholerae*), and membrane transporters (ABC transporters). **(B)** Stacked barplot displaying the distribution of these pathways across individual treatments (A: CTRL, B: CIP, C: PET, D: CIP + PET).

Functional profiles inferred via Tax4Fun (mapping 16S rRNA sequences to SILVA and KEGG Orthologs) revealed dominant pathways including ABC transporters (43.1%), biosynthesis of antibiotics (30.5%), and β-lactam resistance (5.5%) (Figure 6A). Other functions, through less abundant, included biofilm formation in *Pseudomonas aeruginosa* (5.7%), *Escherichia coli* (5.3%), *Vibrio cholerae* (4.1%), and antibiotic resistance pathways for vancomycin (3.3%), CAMP-mediated (0.4%), platinum drugs (0.5%), antifolate (0.4%), and vancomycin biosynthesis (0.3%). Figure 6B shows treatment-specific differences: control (A) displayed 42% ABC transporters and 31% antibiotic biosynthesis; biofilm formation pathways (~15%) and resistance mechanisms (~9%) were also prominent. CIP (B) increased β-lactam (~6.5%) and vancomycin resistance (~4%), despite CIP being a fluoroquinolone, reflecting co-selection of multidrug resistance traits, commonly driven by antibiotic exposure (Martínez et al., 2015; von Wintersdorff et al., 2016). PET (C) mirrored control but showed slight increases in biofilm-related functions (~17%), particularly those related to *P. aeruginosa*, supporting the role of microplastics as biofilm substrate (Zettler et al., 2013). Combined CIP + PET treatment (D) enhanced biofilm functions (~20%), with notable contributions from *P. aeruginosa* and *E. coli*, and antibiotic resistance (~15%), suggesting that microplastics act as hotspots for resistant biofilm communities, facilitating persistence and spread of resistant genes (Zheng et al., 2023). ABC transporters and antibiotic biosynthesis consistently represented ~70% of predicted functions, indicating a stable microbial core engaged in substrate uptake and secondary metabolism, likely foundational ecological processes supporting community stability under stress (Glavinas et al., 2004; Crits-Christoph et al., 2021).

*Post-hoc* Tukey HSD test (Supplementary Table S6; Supplementary Figure S2) confirmed significant differences among treatments in resistance to β-lactams, vancomycin, antifolates, and biofilm formation (notably *P. aeruginosa* and *Vibrio* spp.), corroborating functional predictions. Further validation through metagenomics or cultivation-based assays is necessary to confirm these inferred functionalities.

#### 3.2.3 ARGs abundance

**Figure 7:**
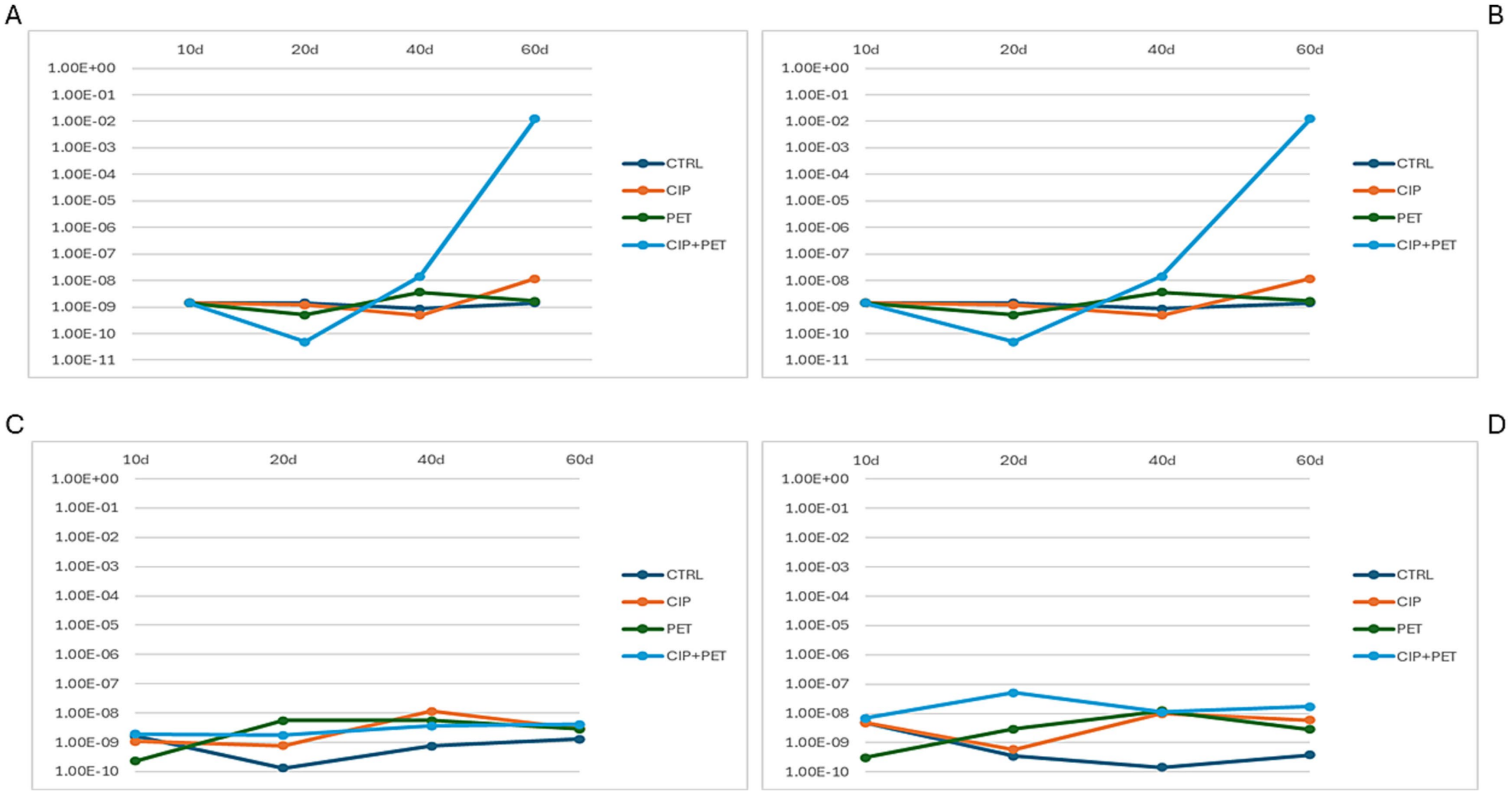
Quantification of CIP resistance genes across four experimental conditions (CTRL, CIP, PET, CIP + PET) and four incubation time points (10 days, 20 days, 40 days and 60 days). Panels show relative abundances of **(A)** qnrA, **(B)** qnrB, **(C)** qnrC, and **(D)** qnrS, highlighting the temporal dynamics and treatment-specific variations in the presence of plasmid-mediated quinolone resistance (PMQR) genes.

Quantitative PCR detected *qnrA*, *qnrB*, *qnrC*, and *qnrS* genes across all treatments and time points (Figure 7). *qnrA* (Figure 7A) and *qnrB* (Figure 7B) increased markedly (~10^8^-fold) under combined CIP + PET exposure by day 60. CIP and PET alone induced smaller increases (~10^3^–10^5^-fold), while control showed steady declined. *qnrS* (Figure 7D) slightly rose under CIP + PET and PET (~10–100-fold), whereas *qnrC* (Figure 7C) remained stable across treatments. These patterns indicate synergistic enhancement of quinolone resistance genes by CIP and PET. CIP exerts selective pressure, while PET may facilitate biofilm formation and promote conditions favorable to HGT, potentially contributing to resistance dissemination (Ventura et al., 2024). The stable *qnrC* abundance, which lacks an SOS-inducible promoter unlike *qnrB*, likely reflects distinct regulation and limited mobility (Recacha et al., 2017; Briales et al., 2012; Robicsek et al., 2006). The decline in ARGs under controlled conditions supports the metabolic cost hypothesis of maintaining resistance genes in absence of antibiotic pressure (Garoff et al., 2018).

Supporting these observations, the Dunn *post hoc* test following Kruskal-Wallis analysis (Supplementary Table S7) revealed several comparisons with significant or borderline significant differences based on *p*-values. Notably, *qnrA* showed a significant temporal decrease in the combined CIP + PET treatment between 20 and 40 days (D_T2 vs. D_T3, *p* = 0.035), and a marginal difference between CIP alone at 40 days versus the combined treatment at the same time point (B_T3 vs. D_T3, *p* = 0.050). For *qnrC*, significant differences were detected between 20 and 40 days under CIP treatment alone (B_T2 vs. B_T3, *p* = 0.049), as well as borderline differences when comparing control to PET (A_T2 vs. C_T2, *p* = 0.054) or combined treatment (A_T2 vs. D_T2, *p* = 0.054) at 20 days. *qnrS* showed a marginally significant difference between control and combined treatment at 20 days (A_T2 vs. D_T2, *p* = 0.054). No significant differences were found for *qnrB*.

Although this study did not directly assess mechanisms such as horizontal gene transfer or plasmid dynamics, the observed increase in *qnr* gene abundance under CIP and PET exposure likely results from a combination of selective proliferation and potential enhancement of gene transfer processes. PET particles have been shown to promote biofilm formation, which can increase local cell density and gene exchange opportunities (Li et al., 2025). Nevertheless, we cannot exclude that the increased ARG abundance may stem predominantly from the selective expansion of resistant taxa already harboring *qnr* genes, rather than *de novo* horizontal acquisition. Future studies using metagenomics or plasmid-resolved sequencing would be valuable to disentangle these contributions.

### 3.3 Linking PET sorption kinetics to ARGs abundance and phylum-level shifts

**Figure 8:**
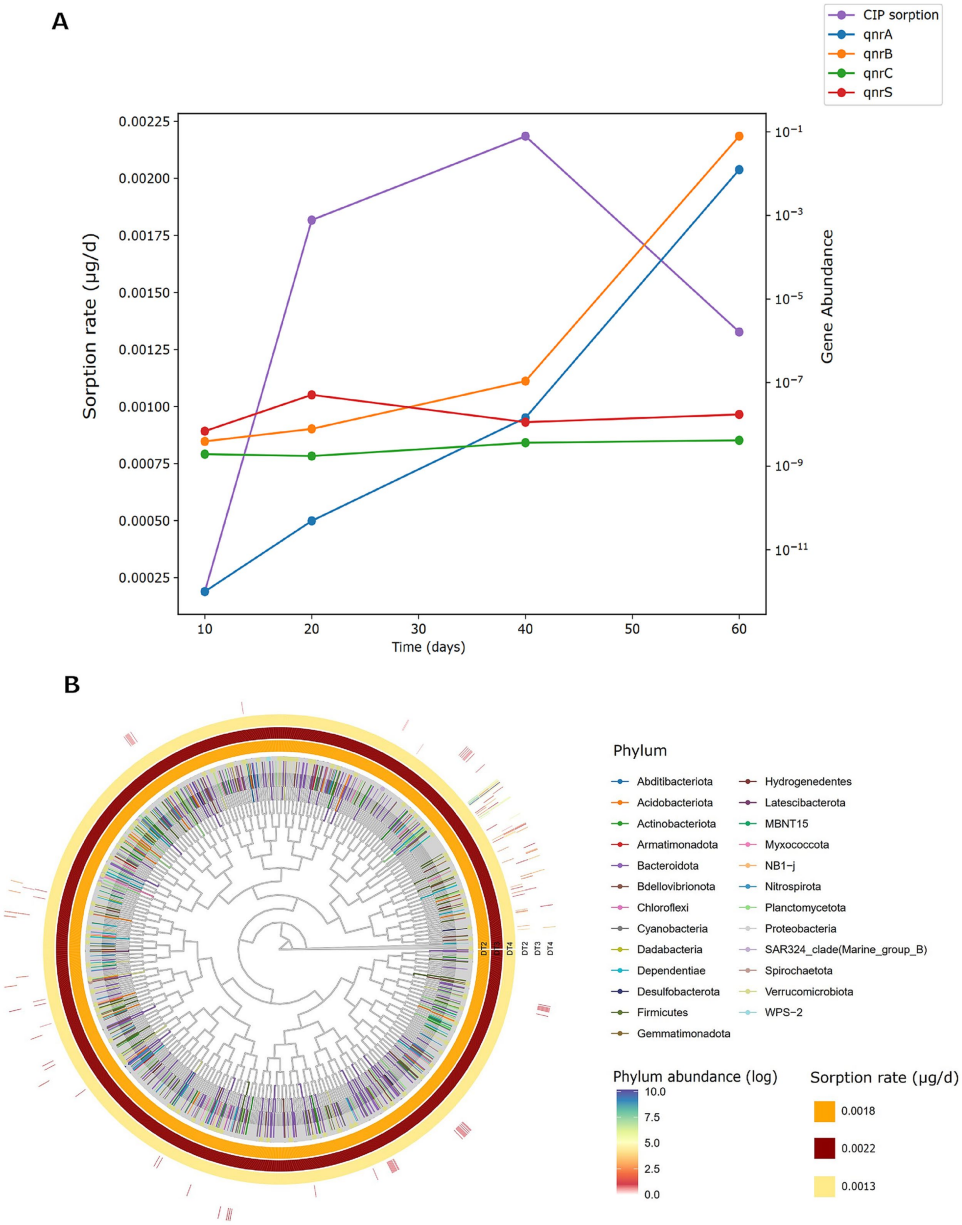
**(A)** Ciprofloxacin sorption rates and relative abundance of resistance genes (qnrA, qnrB, qnrC, qnrS) in sample D (CIP + PET) over 10, 20, 40, and 60 days. Gene abundances are log-transformed. **(B)** Circular phylogenetic tree of bacterial ASVs enriched in sample D across three incubation times (T2 = 20 days, T3 = 40 days, T4 = 60 days) correlating CIP sorption rates. The outer-colored bars represent the log-transformed relative abundance of each phylum, with darker colors indicating higher abundance. Inner rings show sample-specific sorption rates of CIP (μg/day), with color intensity indicating increasing sorption capacity (light yellow = low, dark red = high). Phylum-level taxonomy is color-coded as indicated in the legend.

To elucidate PET’s role as a synergistic vector in antibiotic pollution, and its influence on microbial communities, we correlated CIP sorption kinetics with *qnr* gene abundance under condition D (CIP + PET). CIP sorption rates evaluated using mass balance calculations (Section 3.2.1; Figure 8A), increased steadily over the first 40 days, followed a consistent decrease at day 60. This trend indicates that equilibrium conditions between PET and aqueous phase (Figure 2C) were achieved. The abundance of *qnrA* and *qnrB* genes increased throughout the experiment, with a more pronounced rise after 40 days. This observation can be explained by considering that, once the equilibrium conditions are reached, CIP sorption onto PET becomes negligible, allowing a greater amount of CIP to be retained within the biofilm as it transfers from the aqueous phase. This leads to increased CIP concentrations in the biofilm, thereby enhancing the selection pressure for resistance (Zheng et al., 2023; Ventura et al., 2024). The abundance of *qnrS* showed a modest increase after 20 days, coinciding with the highest observed rise in CIP sorption rate (i.e., 0.0034 mg/day calculated for the 10–20 day interval). In contrast, no significant changes were observed for *qnrC*, likely due to gene-specific regulatory and mobility constraints. Figure 8B displays a circular phylogenetic tree of microbial taxa and CIP sorption rates across 20 (DT2), 40 (DT3), and 60 (DT4) days. Outer bars show phylum relative abundances on a log scale, color-coded by taxonomy; inner rings depict CIP sorption rates (μg/day). Sorption intensity peaks at DT3 (dark red), then declines at DT4 (light yellow). Twenty-five phyla were identified. Dominant phyla included *Proteobacteria* (9.26 × 10^2^), *Bacteroidota* (4.33 × 10^1^), *Firmicutes* (3.71 × 10^1^), *Dependentiae* (2.85 × 10^1^), and *Actinobacteriota* (1.11 × 10^1^). While *Proteobacteria* remained relatively stable, other lineages exhibited temporal shifts: at DT3, coinciding with maximal CIP sorption, *Dependentiae*, *Firmicutes*, *Acidobacteriota*, and *Actinobacteriota* increased, being nearly absent or rare at DT2. At DT4, with lower sorption, *Bacteroidota* and *Planctomycetota* declined markedly, while *Dependentiae* and *Firmicutes* stayed enriched. These taxonomic shifts, linked with chemical sorption data, underscore the interactive role of microplastics and antibiotics in modulating microbial community composition and resistance potential in freshwater ecosystems (Li et al., 2025; Li et al., 2023; Yu et al., 2022).

## 4 Conclusion

This study demonstrates complex interactions between CIP and PET MPs, in affecting microbial communities in alpine spring water microcosms. PET effectively sorbed CIP, reaching equilibrium at 40 days. Sorption kinetics and mass balance suggest combined biosorption and biodegradation determined by biofilm formation. Microbial analyses revealed CIP-induced selective pressures causing pronounced taxonomic shifts, especially in CIP-only and CIP + PET tests, while a resilient core microbiome persisted. PET alone influenced community composition by favoring biofilm-associated taxa, whereas CIP exposure enriched resistant genera (*Janthinobacterium*, *Pedobacter*, *Achromobacter*). NMDS and phylogenetic data confirm CIP as the main driver of restructuring, with PET amplifying effects through surface-mediated ecological mechanisms. qPCR data indicated synergistic enhancement of quinolone resistance genes (*qnrA*, *qnrB*) by combined CIP + PET exposure, highlighting PET’s role as a vector facilitating resistance gene proliferation. In contrast, *qnrC* remained stable, likely due to gene-specific regulatory and mobility constraints. Controls lacking selective agents exhibited ARG decline, consistent with metabolic costs of resistance maintenance. Overall, findings emphasize interconnected antibiotics and microplastics’ intertwined ecological roles in shaping microbial community dynamics and antibiotic resistance dissemination in freshwater environments. Future work should involve more environmentally realistic conditions and integrate metagenomic and cultivation approaches to validate functional inferences and investigate ecological consequences at broader spatiotemporal scales.

## Data Availability

Data were deposited in the NCBI SRA under BioProject accession number PRJNA1241897.
