# Supplementary material for: Combined effects of ciprofloxacin and microplastics on alpine spring water microbiota: evidence from glacier-fed microcosm experiments
Source: Front Microbiol. 2025 Sep 4;16:1654589. doi: 10.3389/fmicb.2025.1654589 (PMC12443835; doi:10.3389/fmicb.2025.1654589)
Supplement: Supplementary file 1 [file Data_Sheet_1.docx]

Supplementary Material

# **Supplementary Figures and Tables**

| **MP** | **T** | **Time (*eq)** | **Speed** | **PWR** | **C_w_** | **Sorption efficiency** | **Reference** |
| --- | --- | --- | --- | --- | --- | --- | --- |
|  | **°C** | **(d)** | **(rpm)** | **(g/L)** | **(mg/L)** | **(%)** |  |
| PS  PVC | 25 | 2 | 150 | 0.4 | 10 | PS (~10)  PS aged (~22)  PVC (~12)  PVC aged ~13) | Liu et al. (2019) |
| PP  LDPE  HDPE  PVC | 21 | 1 | 300 | 100 | 0.5 | PP<PVC<HDPE<LDPE | Puckowski et al. (2021) |
| PE  PS  PP  PA  PVC | 25 | 4 | 180 | 4 | 0.5-15 | PVC<PS<PP<PE<PA | Li et al. (2018) |
| PE | - | 0.125 | 150 | 2 | 25 | >0 | Atugoda et al. (2020) |
| PE  PS  PVC  PET | 25 | 7 | 30 | - | 0.1-20 | PVC<PE<PS<PET | Lin et al. (2020) |
| PS | 25 | 1 | 200 | 0.1 | 0.2-0.8 | 4-50% | Yilimulati et al. (2021) |
| PS  PP  PA | 25 | 2 | 150 | 0.2 | 2-12 | PS<PP<PA | Liu et al. (2022) |
| PLA  PE | 25 | 4 | 180 | 5 | 3 | 21-75%  aged PLA>PLA>aged PE>PE | Liang et al. (2023) |
| PA  PVC | 25 | 2 | 180 | 2 | 3.3 | 60-87 | Tong et al. (2023) |
| PS | 25 | 0.5 | 180 | 1 | 2 | 62.5% | Gao et al. (2024) |
| PE | 25 | 2 | 150 | 0.25-0.75 | 10 | 8% | Lv et al. (2023) |
| PET | 25-40 | 0.5 | 100 | 10 | 5-50 | 94-96% | Enyoh et al. (2023) |
| PS | 25 | 3 | 150 | 1 | 10 | 15% | Liu et al. (2023) |
| PP |  | 2 |  | 4 | 1-40 | 10% | Chen et al. (2023) |
| PE  PLA | 25 | 2 | 200 | 1 | 5 | 8% | Wu et al. (2023) |
| PS | 25 | 2 | 200 | 5 | 5 | 10-50% | Li et al. (2024) |
| PS | room | 0.25 | 120 | 0.2 | 20 | <1% | Gong et al. (2024) |
| TWP | 25 | 5 | 150 | 2.5 | 10 | 6-22% | Xu et al. (2025) |

**Supplementary Table S1**

Sorption studies of MP-CIP-water combination (HDPE: high-density polyethylene; LDPE: low-density polyethylene; PA: polyamide; PE: polyethylene; PET: polyethylene terephthalate; PLA: polylactic acid; PP: polypropylene; PS: polystyrene; PVC: polyvinyl chloride; TWP: Tire wear particles; PWR: polymer-to-water ratio, C_w_: aqueous concentration).

| **Element** | **d_D_** | **d_P_** | **d_H_** |
| --- | --- | --- | --- |
|  | MPa^1/2^ | MPa^1/2^ | MPa^1/2^ |
| CIP | 21.1 | 5.7 | 10.4 |
| HDPE | 18.0 | 0.0 | 2.0 |
| HDPE/LDPE | 17.5 | 4.3 | 8.3 |
| LDPE | 16.5 | 5.9 | 4.1 |
| LDPE | 16.5 | 4.5 | 0.5 |
| LDPE | 15.3 | 5.3 | 2.5 |
| PA | 18.2 | 5.1 | 13.7 |
| PA | 17.0 | 3.4 | 10.6 |
| PA | 17.4 | 9.8 | 14.6 |
| PA | 17 | 4.4 | 10.6 |
| PA | 18.5 | 8.1 | 9.1 |
| PA | 18.2 | 8.8 | 10.8 |
| PA | 16.0 | 11.0 | 24.0 |
| PE | 17.6 | 0.0 | 2.0 |
| PE | 16.9 | 3.3 | 4.1 |
| PE | 17.1 | 3.1 | 5.2 |
| PE | 24.1 | 14.9 | 0.3 |
| PET | 18.2 | 7.3 | 7.9 |
| PET | 18.2 | 6.4 | 6.6 |
| PET | 19.1 | 6.3 | 9.1 |
| PET | 18.0 | 6.2 | 6.2 |
| PET | 18.0 | 3.0 | 4.0 |
| PP | 18.0 | 0.0 | 0.0 |
| PP | 18.0 | 0.0 | 1.0 |
| PP | 17.2 | 5.6 | -0.4 |
| PS | 18.7 | 5.9 | 3.5 |
| PS | 22.3 | 5.8 | 4.3 |
| PS | 18.5 | 4.5 | 2.9 |
| PVC | 18.6 | 8.8 | 5.8 |
| PVC | 18.4 | 6.6 | 8.0 |
| PVC | 17.6 | 7.8 | 3.4 |
| PVC | 16.1 | 5.1 | 5.9 |
| PVC | 14.9 | 11.1 | 3.8 |
| PVC | 24.4 | 4.9 | 9.9 |

**Supplementary Table S2**

Hansen Solubility Parameters (HSP) for CIP (from Silva et al., 2018) and the common type of MPs (from Hansen, 2007). MPs data are related to different grades of the same polymers.

| Adonis |  |  |  |  |  |
| --- | --- | --- | --- | --- | --- |
|  |  |  |  |  |  |
|  | Df | Sum Sq | R^2^ | F | Pr(>F) |
| Model | 3 | 1.791117 | 0.722893 | 17.39139 | 0.001 |
| Residual | 20 | 0.686592 | 0.277107 | NA | NA |
| Total | 23 | 2.477709 | 1 | NA | NA |
|  |  |  |  |  |  |
| Beta dispersion ANOVA | | |  |  |  |
|  |  |  |  |  |  |
|  | Df | Sum Sq | Mean Sq | F value | Pr(>F) |
| Groups | 3 | 0.039073 | 0.013024 | 1.078633 | 0.38068 |
| Residuals | 20 | 0.241498 | 0.012075 | NA | NA |
|  |  |  |  |  |  |
| Beta dispersion permutest | | |  |  |  |
|  |  |  |  |  |  |
|  | Df | Sum Sq | Mean Sq | F | N.Perm |
| Groups | 3 | 0.039073 | 0.013024 | 1.078633 | 999 |
| Residuals | 20 | 0.241498 | 0.012075 | NA | NA |

**Supplementary Table S3**

PERMANOVA analysis based on Bray–Curtis dissimilarities (999 permutations), ANOVA beta dispersion and permutation test of microbial community composition.

| CIP | CIP+PET | CTRL | PET | index | stat | P_value | Taxa |  |  |  |  |  |  |  |  |  |  |
| --- | --- | --- | --- | --- | --- | --- | --- | --- | --- | --- | --- | --- | --- | --- | --- | --- | --- |
| 0 | 0 | 1 | 1 | 10 | 0.99 | 0.006 | d__Bacteria.p__Planctomycetota.c__Planctomycetes.o__Planctomycetales.f__uncultured.g__uncultured | | | | | | | | | |  |
| 1 | 1 | 0 | 0 | 5 | 1 | 0.008 | d__Bacteria.p__Bacteroidota.c__Bacteroidia.o__Chitinophagales.f__Chitinophagaceae.g__Chitinophaga | | | | | | | | | |  |
| 0 | 0 | 1 | 1 | 10 | 0.99 | 0.008 | d__Bacteria.p__Bacteroidota.c__Bacteroidia.o__Chitinophagales.f__Chitinophagaceae.g__Sediminibacterium | | | | | | | | | | |
| 0 | 0 | 1 | 1 | 10 | 0.99 | 0.008 | d__Bacteria.p__Bacteroidota.c__Kapabacteria.o__Kapabacteriales.f__Kapabacteriales.g__Kapabacteriales | | | | | | | | | |  |
| 0 | 0 | 1 | 1 | 10 | 0.99 | 0.008 | d__Bacteria.p__Firmicutes.c__Clostridia.o__Clostridiales.f__Clostridiaceae.g__Clostridium_sensu_stricto_10 | | | | | | | | | | |
| 0 | 0 | 1 | 1 | 10 | 0.99 | 0.008 | d__Bacteria.p__Proteobacteria.c__Alphaproteobacteria.o__Rhizobiales.f__Rhizobiaceae.__ | | | | | | | | |  |  |
| 1 | 0 | 1 | 1 | 13 | 1 | 0.014 | d__Bacteria.p__Verrucomicrobiota.c__Verrucomicrobiae.o__Verrucomicrobiales.f__Rubritaleaceae.g__Luteolibacter | | | | | | | | | | |
| 0 | 0 | 1 | 0 | 3 | 0.98 | 0.039 | d__Bacteria.p__Bacteroidota.c__Bacteroidia.o__Sphingobacteriales.f__KD3.93.g__KD3.93 | | | | | | | | |  |  |

**Supplementary Table S4**

Indicator Species Analysis of ASVs significantly associated with specific treatments (*p* < 0.05).

| Comparison | Z | p-value | Index |
| --- | --- | --- | --- |
| A_T2 - A_T3 | -0.196 | 0.845 | Taxa |
| A_T2 - A_T4 | -0.981 | 0.327 | Taxa |
| A_T3 - A_T4 | -0.784 | 0.433 | Taxa |
| A_T2 - B_T2 | 0.392 | 0.695 | Taxa |
| A_T3 - B_T2 | 0.588 | 0.556 | Taxa |
| A_T4 - B_T2 | 1.373 | 0.170 | Taxa |
| A_T2 - B_T3 | 0.588 | 0.556 | Taxa |
| A_T3 - B_T3 | 0.784 | 0.433 | Taxa |
| A_T4 - B_T3 | 1.569 | 0.117 | Taxa |
| B_T2 - B_T3 | 0.196 | 0.845 | Taxa |
| A_T2 - B_T4 | 1.177 | 0.239 | Taxa |
| A_T3 - B_T4 | 1.373 | 0.170 | Taxa |
| A_T4 - B_T4 | 2.157 | 0.031 | Taxa |
| B_T2 - B_T4 | 0.784 | 0.433 | Taxa |
| B_T3 - B_T4 | 0.588 | 0.556 | Taxa |
| A_T2 - C_T2 | -0.392 | 0.695 | Taxa |
| A_T3 - C_T2 | -0.196 | 0.845 | Taxa |
| A_T4 - C_T2 | 0.588 | 0.556 | Taxa |
| B_T2 - C_T2 | -0.784 | 0.433 | Taxa |
| B_T3 - C_T2 | -0.981 | 0.327 | Taxa |
| B_T4 - C_T2 | -1.569 | 0.117 | Taxa |
| A_T2 - C_T3 | -0.588 | 0.556 | Taxa |
| A_T3 - C_T3 | -0.392 | 0.695 | Taxa |
| A_T4 - C_T3 | 0.392 | 0.695 | Taxa |
| B_T2 - C_T3 | -0.981 | 0.327 | Taxa |
| B_T3 - C_T3 | -1.177 | 0.239 | Taxa |
| B_T4 - C_T3 | -1.765 | 0.078 | Taxa |
| C_T2 - C_T3 | -0.196 | 0.845 | Taxa |
| A_T2 - C_T4 | -0.784 | 0.433 | Taxa |
| A_T3 - C_T4 | -0.588 | 0.556 | Taxa |
| A_T4 - C_T4 | 0.196 | 0.845 | Taxa |
| B_T2 - C_T4 | -1.177 | 0.239 | Taxa |
| B_T3 - C_T4 | -1.373 | 0.170 | Taxa |
| B_T4 - C_T4 | -1.961 | 0.050 | Taxa |
| C_T2 - C_T4 | -0.392 | 0.695 | Taxa |
| C_T3 - C_T4 | -0.196 | 0.845 | Taxa |
| A_T2 - D_T2 | 0.981 | 0.327 | Taxa |
| A_T3 - D_T2 | 1.177 | 0.239 | Taxa |
| A_T4 - D_T2 | 1.961 | 0.050 | Taxa |
| B_T2 - D_T2 | 0.588 | 0.556 | Taxa |
| B_T3 - D_T2 | 0.392 | 0.695 | Taxa |
| B_T4 - D_T2 | -0.196 | 0.845 | Taxa |
| C_T2 - D_T2 | 1.373 | 0.170 | Taxa |
| C_T3 - D_T2 | 1.569 | 0.117 | Taxa |
| C_T4 - D_T2 | 1.765 | 0.078 | Taxa |
| A_T2 - D_T3 | 0.196 | 0.845 | Taxa |
| A_T3 - D_T3 | 0.392 | 0.695 | Taxa |
| A_T4 - D_T3 | 1.177 | 0.239 | Taxa |
| B_T2 - D_T3 | -0.196 | 0.845 | Taxa |
| B_T3 - D_T3 | -0.392 | 0.695 | Taxa |
| B_T4 - D_T3 | -0.981 | 0.327 | Taxa |
| C_T2 - D_T3 | 0.588 | 0.556 | Taxa |
| C_T3 - D_T3 | 0.784 | 0.433 | Taxa |
| C_T4 - D_T3 | 0.981 | 0.327 | Taxa |
| D_T2 - D_T3 | -0.784 | 0.433 | Taxa |
| A_T2 - D_T4 | 0.784 | 0.433 | Taxa |
| A_T3 - D_T4 | 0.981 | 0.327 | Taxa |
| A_T4 - D_T4 | 1.765 | 0.078 | Taxa |
| B_T2 - D_T4 | 0.392 | 0.695 | Taxa |
| B_T3 - D_T4 | 0.196 | 0.845 | Taxa |
| B_T4 - D_T4 | -0.392 | 0.695 | Taxa |
| C_T2 - D_T4 | 1.177 | 0.239 | Taxa |
| C_T3 - D_T4 | 1.373 | 0.170 | Taxa |
| C_T4 - D_T4 | 1.569 | 0.117 | Taxa |
| D_T2 - D_T4 | -0.196 | 0.845 | Taxa |
| D_T3 - D_T4 | 0.588 | 0.556 | Taxa |
| A_T2 - A_T3 | -0.491 | 0.623 | Shannon |
| A_T2 - A_T4 | -0.786 | 0.432 | Shannon |
| A_T3 - A_T4 | -0.295 | 0.768 | Shannon |
| A_T2 - B_T2 | 0.982 | 0.326 | Shannon |
| A_T3 - B_T2 | 1.473 | 0.141 | Shannon |
| A_T4 - B_T2 | 1.768 | 0.077 | Shannon |
| A_T2 - B_T3 | 1.179 | 0.238 | Shannon |
| A_T3 - B_T3 | 1.670 | 0.095 | Shannon |
| A_T4 - B_T3 | 1.965 | 0.049 | Shannon |
| B_T2 - B_T3 | 0.196 | 0.844 | Shannon |
| A_T2 - B_T4 | 0.786 | 0.432 | Shannon |
| A_T3 - B_T4 | 1.277 | 0.202 | Shannon |
| A_T4 - B_T4 | 1.572 | 0.116 | Shannon |
| B_T2 - B_T4 | -0.196 | 0.844 | Shannon |
| B_T3 - B_T4 | -0.393 | 0.694 | Shannon |
| A_T2 - C_T2 | -0.196 | 0.844 | Shannon |
| A_T3 - C_T2 | 0.295 | 0.768 | Shannon |
| A_T4 - C_T2 | 0.589 | 0.556 | Shannon |
| B_T2 - C_T2 | -1.179 | 0.238 | Shannon |
| B_T3 - C_T2 | -1.375 | 0.169 | Shannon |
| B_T4 - C_T2 | -0.982 | 0.326 | Shannon |
| A_T2 - C_T3 | -0.491 | 0.623 | Shannon |
| A_T3 - C_T3 | 0.000 | 1.000 | Shannon |
| A_T4 - C_T3 | 0.295 | 0.768 | Shannon |
| B_T2 - C_T3 | -1.473 | 0.141 | Shannon |
| B_T3 - C_T3 | -1.670 | 0.095 | Shannon |
| B_T4 - C_T3 | -1.277 | 0.202 | Shannon |
| C_T2 - C_T3 | -0.295 | 0.768 | Shannon |
| A_T2 - C_T4 | -0.982 | 0.326 | Shannon |
| A_T3 - C_T4 | -0.491 | 0.623 | Shannon |
| A_T4 - C_T4 | -0.196 | 0.844 | Shannon |
| B_T2 - C_T4 | -1.965 | 0.049 | Shannon |
| B_T3 - C_T4 | -2.161 | 0.031 | Shannon |
| B_T4 - C_T4 | -1.768 | 0.077 | Shannon |
| C_T2 - C_T4 | -0.786 | 0.432 | Shannon |
| C_T3 - C_T4 | -0.491 | 0.623 | Shannon |
| A_T2 - D_T2 | 0.393 | 0.694 | Shannon |
| A_T3 - D_T2 | 0.884 | 0.377 | Shannon |
| A_T4 - D_T2 | 1.179 | 0.238 | Shannon |
| B_T2 - D_T2 | -0.589 | 0.556 | Shannon |
| B_T3 - D_T2 | -0.786 | 0.432 | Shannon |
| B_T4 - D_T2 | -0.393 | 0.694 | Shannon |
| C_T2 - D_T2 | 0.589 | 0.556 | Shannon |
| C_T3 - D_T2 | 0.884 | 0.377 | Shannon |
| C_T4 - D_T2 | 1.375 | 0.169 | Shannon |
| A_T2 - D_T3 | 0.589 | 0.556 | Shannon |
| A_T3 - D_T3 | 1.081 | 0.280 | Shannon |
| A_T4 - D_T3 | 1.375 | 0.169 | Shannon |
| B_T2 - D_T3 | -0.393 | 0.694 | Shannon |
| B_T3 - D_T3 | -0.589 | 0.556 | Shannon |
| B_T4 - D_T3 | -0.196 | 0.844 | Shannon |
| C_T2 - D_T3 | 0.786 | 0.432 | Shannon |
| C_T3 - D_T3 | 1.081 | 0.280 | Shannon |
| C_T4 - D_T3 | 1.572 | 0.116 | Shannon |
| D_T2 - D_T3 | 0.196 | 0.844 | Shannon |
| A_T2 - D_T4 | 0.196 | 0.844 | Shannon |
| A_T3 - D_T4 | 0.688 | 0.492 | Shannon |
| A_T4 - D_T4 | 0.982 | 0.326 | Shannon |
| B_T2 - D_T4 | -0.786 | 0.432 | Shannon |
| B_T3 - D_T4 | -0.982 | 0.326 | Shannon |
| B_T4 - D_T4 | -0.589 | 0.556 | Shannon |
| C_T2 - D_T4 | 0.393 | 0.694 | Shannon |
| C_T3 - D_T4 | 0.688 | 0.492 | Shannon |
| C_T4 - D_T4 | 1.179 | 0.238 | Shannon |
| D_T2 - D_T4 | -0.196 | 0.844 | Shannon |
| D_T3 - D_T4 | -0.393 | 0.694 | Shannon |
| A_T2 - A_T3 | -0.700 | 0.484 | Simpson |
| A_T2 - A_T4 | -0.700 | 0.484 | Simpson |
| A_T3 - A_T4 | 0.000 | 1.000 | Simpson |
| A_T2 - B_T2 | 0.800 | 0.424 | Simpson |
| A_T3 - B_T2 | 1.500 | 0.134 | Simpson |
| A_T4 - B_T2 | 1.500 | 0.134 | Simpson |
| A_T2 - B_T3 | 1.200 | 0.230 | Simpson |
| A_T3 - B_T3 | 1.900 | 0.057 | Simpson |
| A_T4 - B_T3 | 1.900 | 0.057 | Simpson |
| B_T2 - B_T3 | 0.400 | 0.689 | Simpson |
| A_T2 - B_T4 | 1.000 | 0.317 | Simpson |
| A_T3 - B_T4 | 1.700 | 0.089 | Simpson |
| A_T4 - B_T4 | 1.700 | 0.089 | Simpson |
| B_T2 - B_T4 | 0.200 | 0.841 | Simpson |
| B_T3 - B_T4 | -0.200 | 0.841 | Simpson |
| A_T2 - C_T2 | -0.700 | 0.484 | Simpson |
| A_T3 - C_T2 | 0.000 | 1.000 | Simpson |
| A_T4 - C_T2 | 0.000 | 1.000 | Simpson |
| B_T2 - C_T2 | -1.500 | 0.134 | Simpson |
| B_T3 - C_T2 | -1.900 | 0.057 | Simpson |
| B_T4 - C_T2 | -1.700 | 0.089 | Simpson |
| A_T2 - C_T3 | -0.200 | 0.841 | Simpson |
| A_T3 - C_T3 | 0.500 | 0.617 | Simpson |
| A_T4 - C_T3 | 0.500 | 0.617 | Simpson |
| B_T2 - C_T3 | -1.000 | 0.317 | Simpson |
| B_T3 - C_T3 | -1.400 | 0.162 | Simpson |
| B_T4 - C_T3 | -1.200 | 0.230 | Simpson |
| C_T2 - C_T3 | 0.500 | 0.617 | Simpson |
| A_T2 - C_T4 | -0.700 | 0.484 | Simpson |
| A_T3 - C_T4 | 0.000 | 1.000 | Simpson |
| A_T4 - C_T4 | 0.000 | 1.000 | Simpson |
| B_T2 - C_T4 | -1.500 | 0.134 | Simpson |
| B_T3 - C_T4 | -1.900 | 0.057 | Simpson |
| B_T4 - C_T4 | -1.700 | 0.089 | Simpson |
| C_T2 - C_T4 | 0.000 | 1.000 | Simpson |
| C_T3 - C_T4 | -0.500 | 0.617 | Simpson |
| A_T2 - D_T2 | 0.200 | 0.841 | Simpson |
| A_T3 - D_T2 | 0.900 | 0.368 | Simpson |
| A_T4 - D_T2 | 0.900 | 0.368 | Simpson |
| B_T2 - D_T2 | -0.600 | 0.549 | Simpson |
| B_T3 - D_T2 | -1.000 | 0.317 | Simpson |
| B_T4 - D_T2 | -0.800 | 0.424 | Simpson |
| C_T2 - D_T2 | 0.900 | 0.368 | Simpson |
| C_T3 - D_T2 | 0.400 | 0.689 | Simpson |
| C_T4 - D_T2 | 0.900 | 0.368 | Simpson |
| A_T2 - D_T3 | 0.500 | 0.617 | Simpson |
| A_T3 - D_T3 | 1.200 | 0.230 | Simpson |
| A_T4 - D_T3 | 1.200 | 0.230 | Simpson |
| B_T2 - D_T3 | -0.300 | 0.764 | Simpson |
| B_T3 - D_T3 | -0.700 | 0.484 | Simpson |
| B_T4 - D_T3 | -0.500 | 0.617 | Simpson |
| C_T2 - D_T3 | 1.200 | 0.230 | Simpson |
| C_T3 - D_T3 | 0.700 | 0.484 | Simpson |
| C_T4 - D_T3 | 1.200 | 0.230 | Simpson |
| D_T2 - D_T3 | 0.300 | 0.764 | Simpson |
| A_T2 - D_T4 | 0.500 | 0.617 | Simpson |
| A_T3 - D_T4 | 1.200 | 0.230 | Simpson |
| A_T4 - D_T4 | 1.200 | 0.230 | Simpson |
| B_T2 - D_T4 | -0.300 | 0.764 | Simpson |
| B_T3 - D_T4 | -0.700 | 0.484 | Simpson |
| B_T4 - D_T4 | -0.500 | 0.617 | Simpson |
| C_T2 - D_T4 | 1.200 | 0.230 | Simpson |
| C_T3 - D_T4 | 0.700 | 0.484 | Simpson |
| C_T4 - D_T4 | 1.200 | 0.230 | Simpson |
| D_T2 - D_T4 | 0.300 | 0.764 | Simpson |
| D_T3 - D_T4 | 0.000 | 1.000 | Simpson |
| A_T2 - A_T3 | -0.982 | 0.326 | Evenness |
| A_T2 - A_T4 | -0.295 | 0.768 | Evenness |
| A_T3 - A_T4 | 0.688 | 0.492 | Evenness |
| A_T2 - B_T2 | 0.982 | 0.326 | Evenness |
| A_T3 - B_T2 | 1.965 | 0.049 | Evenness |
| A_T4 - B_T2 | 1.277 | 0.202 | Evenness |
| A_T2 - B_T3 | 1.179 | 0.238 | Evenness |
| A_T3 - B_T3 | 2.161 | 0.031 | Evenness |
| A_T4 - B_T3 | 1.473 | 0.141 | Evenness |
| B_T2 - B_T3 | 0.196 | 0.844 | Evenness |
| A_T2 - B_T4 | 0.589 | 0.556 | Evenness |
| A_T3 - B_T4 | 1.572 | 0.116 | Evenness |
| A_T4 - B_T4 | 0.884 | 0.377 | Evenness |
| B_T2 - B_T4 | -0.393 | 0.694 | Evenness |
| B_T3 - B_T4 | -0.589 | 0.556 | Evenness |
| A_T2 - C_T2 | -0.589 | 0.556 | Evenness |
| A_T3 - C_T2 | 0.393 | 0.694 | Evenness |
| A_T4 - C_T2 | -0.295 | 0.768 | Evenness |
| B_T2 - C_T2 | -1.572 | 0.116 | Evenness |
| B_T3 - C_T2 | -1.768 | 0.077 | Evenness |
| B_T4 - C_T2 | -1.179 | 0.238 | Evenness |
| A_T2 - C_T3 | -0.295 | 0.768 | Evenness |
| A_T3 - C_T3 | 0.688 | 0.492 | Evenness |
| A_T4 - C_T3 | 0.000 | 1.000 | Evenness |
| B_T2 - C_T3 | -1.277 | 0.202 | Evenness |
| B_T3 - C_T3 | -1.473 | 0.141 | Evenness |
| B_T4 - C_T3 | -0.884 | 0.377 | Evenness |
| C_T2 - C_T3 | 0.295 | 0.768 | Evenness |
| A_T2 - C_T4 | -0.786 | 0.432 | Evenness |
| A_T3 - C_T4 | 0.196 | 0.844 | Evenness |
| A_T4 - C_T4 | -0.491 | 0.623 | Evenness |
| B_T2 - C_T4 | -1.768 | 0.077 | Evenness |
| B_T3 - C_T4 | -1.965 | 0.049 | Evenness |
| B_T4 - C_T4 | -1.375 | 0.169 | Evenness |
| C_T2 - C_T4 | -0.196 | 0.844 | Evenness |
| C_T3 - C_T4 | -0.491 | 0.623 | Evenness |
| A_T2 - D_T2 | 0.393 | 0.694 | Evenness |
| A_T3 - D_T2 | 1.375 | 0.169 | Evenness |
| A_T4 - D_T2 | 0.688 | 0.492 | Evenness |
| B_T2 - D_T2 | -0.589 | 0.556 | Evenness |
| B_T3 - D_T2 | -0.786 | 0.432 | Evenness |
| B_T4 - D_T2 | -0.196 | 0.844 | Evenness |
| C_T2 - D_T2 | 0.982 | 0.326 | Evenness |
| C_T3 - D_T2 | 0.688 | 0.492 | Evenness |
| C_T4 - D_T2 | 1.179 | 0.238 | Evenness |
| A_T2 - D_T3 | 0.786 | 0.432 | Evenness |
| A_T3 - D_T3 | 1.768 | 0.077 | Evenness |
| A_T4 - D_T3 | 1.081 | 0.280 | Evenness |
| B_T2 - D_T3 | -0.196 | 0.844 | Evenness |
| B_T3 - D_T3 | -0.393 | 0.694 | Evenness |
| B_T4 - D_T3 | 0.196 | 0.844 | Evenness |
| C_T2 - D_T3 | 1.375 | 0.169 | Evenness |
| C_T3 - D_T3 | 1.081 | 0.280 | Evenness |
| C_T4 - D_T3 | 1.572 | 0.116 | Evenness |
| D_T2 - D_T3 | 0.393 | 0.694 | Evenness |
| A_T2 - D_T4 | 0.196 | 0.844 | Evenness |
| A_T3 - D_T4 | 1.179 | 0.238 | Evenness |
| A_T4 - D_T4 | 0.491 | 0.623 | Evenness |
| B_T2 - D_T4 | -0.786 | 0.432 | Evenness |
| B_T3 - D_T4 | -0.982 | 0.326 | Evenness |
| B_T4 - D_T4 | -0.393 | 0.694 | Evenness |
| C_T2 - D_T4 | 0.786 | 0.432 | Evenness |
| C_T3 - D_T4 | 0.491 | 0.623 | Evenness |
| C_T4 - D_T4 | 0.982 | 0.326 | Evenness |
| D_T2 - D_T4 | -0.196 | 0.844 | Evenness |
| D_T3 - D_T4 | -0.589 | 0.556 | Evenness |

**Supplementary Table S5**

Dunn’s post hoc comparisons of diversity indices.

| **contrast** | **p_value** | **Pathway** |
| --- | --- | --- |
| B-A | 0.32383 | Biosinthesys of vancomycin |
| C-A | 0.687794 | Biosinthesys of vancomycin |
| D-A | 0.731518 | Biosinthesys of vancomycin |
| C-B | 0.039638 | Biosinthesys of vancomycin |
| D-B | 0.84291 | Biosinthesys of vancomycin |
| D-C | 0.150785 | Biosinthesys of vancomycin |
| B-A | 0.910694 | Biosinthesys of antibiotics |
| C-A | 0.98572 | Biosinthesys of antibiotics |
| D-A | 0.583254 | Biosinthesys of antibiotics |
| C-B | 0.985403 | Biosinthesys of antibiotics |
| D-B | 0.898563 | Biosinthesys of antibiotics |
| D-C | 0.734262 | Biosinthesys of antibiotics |
| B-A | 0.005403 | beta-Lactam resistance |
| C-A | 0.988838 | beta-Lactam resistance |
| D-A | 0.0288 | beta-Lactam resistance |
| C-B | 0.005307 | beta-Lactam resistance |
| D-B | 0.701105 | beta-Lactam resistance |
| D-C | 0.032379 | beta-Lactam resistance |
| B-A | 0.008722 | vancomycin resistance |
| C-A | 0.881946 | vancomycin resistance |
| D-A | 0.040816 | vancomycin resistance |
| C-B | 0.018911 | vancomycin resistance |
| D-B | 0.752395 | vancomycin resistance |
| D-C | 0.099364 | vancomycin resistance |
| B-A | 0.073191 | CAMP resistance |
| C-A | 0.74806 | CAMP resistance |
| D-A | 0.043924 | CAMP resistance |
| C-B | 0.277699 | CAMP resistance |
| D-B | 0.986557 | CAMP resistance |
| D-C | 0.170771 | CAMP resistance |
| B-A | 0.005267 | Antifolate resistance |
| C-A | 0.99229 | Antifolate resistance |
| D-A | 0.024851 | Antifolate resistance |
| C-B | 0.004839 | Antifolate resistance |
| D-B | 0.744736 | Antifolate resistance |
| D-C | 0.025813 | Antifolate resistance |
| B-A | 0.287293 | Platinum drug resistance |
| C-A | 0.999169 | Platinum drug resistance |
| D-A | 0.758026 | Platinum drug resistance |
| C-B | 0.282945 | Platinum drug resistance |
| D-B | 0.768844 | Platinum drug resistance |
| D-C | 0.790269 | Platinum drug resistance |
| B-A | 0.581925 | ABC transporters |
| C-A | 0.995069 | ABC transporters |
| D-A | 0.989088 | ABC transporters |
| C-B | 0.388322 | ABC transporters |
| D-B | 0.714699 | ABC transporters |
| D-C | 0.932933 | ABC transporters |
| B-A | 0.018632 | Biofilm formation- *Pseudomonas aeruginosa* |
| C-A | 0.970692 | Biofilm formation- *Pseudomonas aeruginosa* |
| D-A | 0.110386 | Biofilm formation- *Pseudomonas aeruginosa* |
| C-B | 0.005318 | Biofilm formation- *Pseudomonas aeruginosa* |
| D-B | 0.661861 | Biofilm formation- *Pseudomonas aeruginosa* |
| D-C | 0.036397 | Biofilm formation- *Pseudomonas aeruginosa* |
| B-A | 0.988825 | Biofilm formation- *Escherichia coli* |
| C-A | 0.998503 | Biofilm formation- *Escherichia coli* |
| D-A | 0.954919 | Biofilm formation- *Escherichia coli* |
| C-B | 0.998244 | Biofilm formation- *Escherichia coli* |
| D-B | 0.996352 | Biofilm formation- *Escherichia coli* |
| D-C | 0.980311 | Biofilm formation- *Escherichia coli* |
| B-A | 0.029022 | Biofilm formation- *Vibrio spp.* |
| C-A | 0.999923 | Biofilm formation- *Vibrio spp.* |
| D-A | 0.152357 | Biofilm formation- *Vibrio spp.* |
| C-B | 0.020629 | Biofilm formation- *Vibrio spp.* |
| D-B | 0.698825 | Biofilm formation- *Vibrio spp.* |
| D-C | 0.125824 | Biofilm formation- *Vibrio spp.* |

**Supplementary Table S6**

Post-hoc comparisons using the Tukey HSD test on potential functional capabilities of the microbial community from 16S rRNA gene sequences by mapping them to the SILVA reference database and estimating functional profiles based on direct sequence similarity to known genomes.

| Comparison | Z | p-value | Index |
| --- | --- | --- | --- |
| A_T1 - A_T2 | -0.45 | 0.65 | qnrA |
| A_T1 - A_T3 | 0.60 | 0.55 | qnrA |
| A_T2 - A_T3 | 1.06 | 0.29 | qnrA |
| A_T1 - A_T4 | 0.00 | 1.00 | qnrA |
| A_T2 - A_T4 | 0.45 | 0.65 | qnrA |
| A_T3 - A_T4 | -0.60 | 0.55 | qnrA |
| A_T1 - B_T2 | 0.26 | 0.79 | qnrA |
| A_T2 - B_T2 | 0.78 | 0.43 | qnrA |
| A_T3 - B_T2 | -0.44 | 0.66 | qnrA |
| A_T4 - B_T2 | 0.26 | 0.79 | qnrA |
| A_T1 - B_T3 | 0.90 | 0.37 | qnrA |
| A_T2 - B_T3 | 1.36 | 0.17 | qnrA |
| A_T3 - B_T3 | 0.30 | 0.76 | qnrA |
| A_T4 - B_T3 | 0.90 | 0.37 | qnrA |
| B_T2 - B_T3 | 0.78 | 0.43 | qnrA |
| A_T1 - B_T4 | -0.90 | 0.37 | qnrA |
| A_T2 - B_T4 | -0.45 | 0.65 | qnrA |
| A_T3 - B_T4 | -1.51 | 0.13 | qnrA |
| A_T4 - B_T4 | -0.90 | 0.37 | qnrA |
| B_T2 - B_T4 | -1.31 | 0.19 | qnrA |
| B_T3 - B_T4 | -1.81 | 0.07 | qnrA |
| A_T1 - C_T2 | 0.75 | 0.45 | qnrA |
| A_T2 - C_T2 | 1.21 | 0.23 | qnrA |
| A_T3 - C_T2 | 0.15 | 0.88 | qnrA |
| A_T4 - C_T2 | 0.75 | 0.45 | qnrA |
| B_T2 - C_T2 | 0.61 | 0.54 | qnrA |
| B_T3 - C_T2 | -0.15 | 0.88 | qnrA |
| B_T4 - C_T2 | 1.66 | 0.10 | qnrA |
| A_T1 - C_T3 | -0.44 | 0.66 | qnrA |
| A_T2 - C_T3 | 0.09 | 0.93 | qnrA |
| A_T3 - C_T3 | -1.13 | 0.26 | qnrA |
| A_T4 - C_T3 | -0.44 | 0.66 | qnrA |
| B_T2 - C_T3 | -0.85 | 0.39 | qnrA |
| B_T3 - C_T3 | -1.48 | 0.14 | qnrA |
| B_T4 - C_T3 | 0.61 | 0.54 | qnrA |
| C_T2 - C_T3 | -1.31 | 0.19 | qnrA |
| A_T1 - C_T4 | -0.60 | 0.55 | qnrA |
| A_T2 - C_T4 | -0.15 | 0.88 | qnrA |
| A_T3 - C_T4 | -1.21 | 0.23 | qnrA |
| A_T4 - C_T4 | -0.60 | 0.55 | qnrA |
| B_T2 - C_T4 | -0.96 | 0.34 | qnrA |
| B_T3 - C_T4 | -1.51 | 0.13 | qnrA |
| B_T4 - C_T4 | 0.30 | 0.76 | qnrA |
| C_T2 - C_T4 | -1.36 | 0.17 | qnrA |
| C_T3 - C_T4 | -0.26 | 0.79 | qnrA |
| A_T1 - D_T2 | 1.06 | 0.29 | qnrA |
| A_T2 - D_T2 | 1.51 | 0.13 | qnrA |
| A_T3 - D_T2 | 0.45 | 0.65 | qnrA |
| A_T4 - D_T2 | 1.06 | 0.29 | qnrA |
| B_T2 - D_T2 | 0.96 | 0.34 | qnrA |
| B_T3 - D_T2 | 0.15 | 0.88 | qnrA |
| B_T4 - D_T2 | 1.96 | 0.05 | qnrA |
| C_T2 - D_T2 | 0.30 | 0.76 | qnrA |
| C_T3 - D_T2 | 1.65 | 0.10 | qnrA |
| C_T4 - D_T2 | 1.66 | 0.10 | qnrA |
| A_T1 - D_T3 | -1.06 | 0.29 | qnrA |
| A_T2 - D_T3 | -0.60 | 0.55 | qnrA |
| A_T3 - D_T3 | -1.66 | 0.10 | qnrA |
| A_T4 - D_T3 | -1.06 | 0.29 | qnrA |
| B_T2 - D_T3 | -1.48 | 0.14 | qnrA |
| B_T3 - D_T3 | -1.96 | 0.05 | qnrA |
| B_T4 - D_T3 | -0.15 | 0.88 | qnrA |
| C_T2 - D_T3 | -1.81 | 0.07 | qnrA |
| C_T3 - D_T3 | -0.78 | 0.43 | qnrA |
| C_T4 - D_T3 | -0.45 | 0.65 | qnrA |
| D_T2 - D_T3 | -2.11 | 0.03 | qnrA |
| A_T1 - D_T4 | -0.70 | 0.49 | qnrA |
| A_T2 - D_T4 | -0.17 | 0.86 | qnrA |
| A_T3 - D_T4 | -1.39 | 0.16 | qnrA |
| A_T4 - D_T4 | -0.70 | 0.49 | qnrA |
| B_T2 - D_T4 | -1.17 | 0.24 | qnrA |
| B_T3 - D_T4 | -1.74 | 0.08 | qnrA |
| B_T4 - D_T4 | 0.35 | 0.73 | qnrA |
| C_T2 - D_T4 | -1.57 | 0.12 | qnrA |
| C_T3 - D_T4 | -0.32 | 0.75 | qnrA |
| C_T4 - D_T4 | 0.00 | 1.00 | qnrA |
| D_T2 - D_T4 | -1.91 | 0.06 | qnrA |
| D_T3 - D_T4 | 0.52 | 0.60 | qnrA |
| A_T1 - A_T2 | 1.19 | 0.23 | qnrB |
| A_T1 - A_T3 | 1.49 | 0.14 | qnrB |
| A_T2 - A_T3 | 0.30 | 0.77 | qnrB |
| A_T1 - A_T4 | 1.34 | 0.18 | qnrB |
| A_T2 - A_T4 | 0.15 | 0.88 | qnrB |
| A_T3 - A_T4 | -0.15 | 0.88 | qnrB |
| A_T1 - B_T2 | 1.20 | 0.23 | qnrB |
| A_T2 - B_T2 | -0.17 | 0.86 | qnrB |
| A_T3 - B_T2 | -0.51 | 0.61 | qnrB |
| A_T4 - B_T2 | -0.34 | 0.73 | qnrB |
| A_T1 - B_T3 | 0.74 | 0.46 | qnrB |
| A_T2 - B_T3 | -0.45 | 0.66 | qnrB |
| A_T3 - B_T3 | -0.74 | 0.46 | qnrB |
| A_T4 - B_T3 | -0.59 | 0.55 | qnrB |
| B_T2 - B_T3 | -0.34 | 0.73 | qnrB |
| A_T1 - B_T4 | 0.89 | 0.37 | qnrB |
| A_T2 - B_T4 | -0.30 | 0.77 | qnrB |
| A_T3 - B_T4 | -0.59 | 0.55 | qnrB |
| A_T4 - B_T4 | -0.45 | 0.66 | qnrB |
| B_T2 - B_T4 | -0.17 | 0.86 | qnrB |
| B_T3 - B_T4 | 0.15 | 0.88 | qnrB |
| A_T1 - C_T2 | 0.15 | 0.88 | qnrB |
| A_T2 - C_T2 | -1.04 | 0.30 | qnrB |
| A_T3 - C_T2 | -1.34 | 0.18 | qnrB |
| A_T4 - C_T2 | -1.19 | 0.23 | qnrB |
| B_T2 - C_T2 | -1.03 | 0.30 | qnrB |
| B_T3 - C_T2 | -0.59 | 0.55 | qnrB |
| B_T4 - C_T2 | -0.74 | 0.46 | qnrB |
| A_T1 - C_T3 | 1.29 | 0.20 | qnrB |
| A_T2 - C_T3 | -0.09 | 0.93 | qnrB |
| A_T3 - C_T3 | -0.43 | 0.67 | qnrB |
| A_T4 - C_T3 | -0.26 | 0.80 | qnrB |
| B_T2 - C_T3 | 0.11 | 0.92 | qnrB |
| B_T3 - C_T3 | 0.43 | 0.67 | qnrB |
| B_T4 - C_T3 | 0.26 | 0.80 | qnrB |
| C_T2 - C_T3 | 1.11 | 0.26 | qnrB |
| A_T1 - C_T4 | -0.15 | 0.88 | qnrB |
| A_T2 - C_T4 | -1.34 | 0.18 | qnrB |
| A_T3 - C_T4 | -1.63 | 0.10 | qnrB |
| A_T4 - C_T4 | -1.49 | 0.14 | qnrB |
| B_T2 - C_T4 | -1.37 | 0.17 | qnrB |
| B_T3 - C_T4 | -0.89 | 0.37 | qnrB |
| B_T4 - C_T4 | -1.04 | 0.30 | qnrB |
| C_T2 - C_T4 | -0.30 | 0.77 | qnrB |
| C_T3 - C_T4 | -1.46 | 0.14 | qnrB |
| A_T1 - D_T2 | 0.45 | 0.66 | qnrB |
| A_T2 - D_T2 | -0.74 | 0.46 | qnrB |
| A_T3 - D_T2 | -1.04 | 0.30 | qnrB |
| A_T4 - D_T2 | -0.89 | 0.37 | qnrB |
| B_T2 - D_T2 | -0.69 | 0.49 | qnrB |
| B_T3 - D_T2 | -0.30 | 0.77 | qnrB |
| B_T4 - D_T2 | -0.45 | 0.66 | qnrB |
| C_T2 - D_T2 | 0.30 | 0.77 | qnrB |
| C_T3 - D_T2 | -0.77 | 0.44 | qnrB |
| C_T4 - D_T2 | 0.59 | 0.55 | qnrB |
| A_T1 - D_T3 | -0.30 | 0.77 | qnrB |
| A_T2 - D_T3 | -1.49 | 0.14 | qnrB |
| A_T3 - D_T3 | -1.78 | 0.07 | qnrB |
| A_T4 - D_T3 | -1.63 | 0.10 | qnrB |
| B_T2 - D_T3 | -1.54 | 0.12 | qnrB |
| B_T3 - D_T3 | -1.04 | 0.30 | qnrB |
| B_T4 - D_T3 | -1.19 | 0.23 | qnrB |
| C_T2 - D_T3 | -0.45 | 0.66 | qnrB |
| C_T3 - D_T3 | -1.63 | 0.10 | qnrB |
| C_T4 - D_T3 | -0.15 | 0.88 | qnrB |
| D_T2 - D_T3 | -0.74 | 0.46 | qnrB |
| A_T1 - D_T4 | 0.34 | 0.73 | qnrB |
| A_T2 - D_T4 | -1.03 | 0.30 | qnrB |
| A_T3 - D_T4 | -1.37 | 0.17 | qnrB |
| A_T4 - D_T4 | -1.20 | 0.23 | qnrB |
| B_T2 - D_T4 | -1.05 | 0.29 | qnrB |
| B_T3 - D_T4 | -0.51 | 0.61 | qnrB |
| B_T4 - D_T4 | -0.69 | 0.49 | qnrB |
| C_T2 - D_T4 | 0.17 | 0.86 | qnrB |
| C_T3 - D_T4 | -1.16 | 0.25 | qnrB |
| C_T4 - D_T4 | 0.51 | 0.61 | qnrB |
| D_T2 - D_T4 | -0.17 | 0.86 | qnrB |
| D_T3 - D_T4 | 0.69 | 0.49 | qnrB |
| A_T1 - A_T2 | 0.89 | 0.37 | qnrC |
| A_T1 - A_T3 | 0.59 | 0.55 | qnrC |
| A_T2 - A_T3 | -0.30 | 0.77 | qnrC |
| A_T1 - A_T4 | 0.15 | 0.88 | qnrC |
| A_T2 - A_T4 | -0.74 | 0.46 | qnrC |
| A_T3 - A_T4 | -0.45 | 0.66 | qnrC |
| A_T1 - B_T2 | 0.43 | 0.67 | qnrC |
| A_T2 - B_T2 | -0.60 | 0.55 | qnrC |
| A_T3 - B_T2 | -0.26 | 0.80 | qnrC |
| A_T4 - B_T2 | 0.26 | 0.80 | qnrC |
| A_T1 - B_T3 | -1.34 | 0.18 | qnrC |
| A_T2 - B_T3 | -2.23 | 0.03 | qnrC |
| A_T3 - B_T3 | -1.93 | 0.05 | qnrC |
| A_T4 - B_T3 | -1.49 | 0.14 | qnrC |
| B_T2 - B_T3 | -1.97 | 0.05 | qnrC |
| A_T1 - B_T4 | -0.59 | 0.55 | qnrC |
| A_T2 - B_T4 | -1.49 | 0.14 | qnrC |
| A_T3 - B_T4 | -1.19 | 0.23 | qnrC |
| A_T4 - B_T4 | -0.74 | 0.46 | qnrC |
| B_T2 - B_T4 | -1.11 | 0.26 | qnrC |
| B_T3 - B_T4 | 0.74 | 0.46 | qnrC |
| A_T1 - C_T2 | -1.04 | 0.30 | qnrC |
| A_T2 - C_T2 | -1.93 | 0.05 | qnrC |
| A_T3 - C_T2 | -1.63 | 0.10 | qnrC |
| A_T4 - C_T2 | -1.19 | 0.23 | qnrC |
| B_T2 - C_T2 | -1.63 | 0.10 | qnrC |
| B_T3 - C_T2 | 0.30 | 0.77 | qnrC |
| B_T4 - C_T2 | -0.45 | 0.66 | qnrC |
| A_T1 - C_T3 | -0.26 | 0.80 | qnrC |
| A_T2 - C_T3 | -1.29 | 0.20 | qnrC |
| A_T3 - C_T3 | -0.94 | 0.35 | qnrC |
| A_T4 - C_T3 | -0.43 | 0.67 | qnrC |
| B_T2 - C_T3 | -0.84 | 0.40 | qnrC |
| B_T3 - C_T3 | 1.29 | 0.20 | qnrC |
| B_T4 - C_T3 | 0.43 | 0.67 | qnrC |
| C_T2 - C_T3 | 0.94 | 0.35 | qnrC |
| A_T1 - C_T4 | -0.45 | 0.66 | qnrC |
| A_T2 - C_T4 | -1.34 | 0.18 | qnrC |
| A_T3 - C_T4 | -1.04 | 0.30 | qnrC |
| A_T4 - C_T4 | -0.59 | 0.55 | qnrC |
| B_T2 - C_T4 | -0.94 | 0.35 | qnrC |
| B_T3 - C_T4 | 0.89 | 0.37 | qnrC |
| B_T4 - C_T4 | 0.15 | 0.88 | qnrC |
| C_T2 - C_T4 | 0.59 | 0.55 | qnrC |
| C_T3 - C_T4 | -0.26 | 0.80 | qnrC |
| A_T1 - D_T2 | -0.15 | 0.88 | qnrC |
| A_T2 - D_T2 | -1.04 | 0.30 | qnrC |
| A_T3 - D_T2 | -0.74 | 0.46 | qnrC |
| A_T4 - D_T2 | -0.30 | 0.77 | qnrC |
| B_T2 - D_T2 | -0.60 | 0.55 | qnrC |
| B_T3 - D_T2 | 1.19 | 0.23 | qnrC |
| B_T4 - D_T2 | 0.45 | 0.66 | qnrC |
| C_T2 - D_T2 | 0.89 | 0.37 | qnrC |
| C_T3 - D_T2 | 0.09 | 0.93 | qnrC |
| C_T4 - D_T2 | 0.30 | 0.77 | qnrC |
| A_T1 - D_T3 | -0.74 | 0.46 | qnrC |
| A_T2 - D_T3 | -1.63 | 0.10 | qnrC |
| A_T3 - D_T3 | -1.34 | 0.18 | qnrC |
| A_T4 - D_T3 | -0.89 | 0.37 | qnrC |
| B_T2 - D_T3 | -1.29 | 0.20 | qnrC |
| B_T3 - D_T3 | 0.59 | 0.55 | qnrC |
| B_T4 - D_T3 | -0.15 | 0.88 | qnrC |
| C_T2 - D_T3 | 0.30 | 0.77 | qnrC |
| C_T3 - D_T3 | -0.60 | 0.55 | qnrC |
| C_T4 - D_T3 | -0.30 | 0.77 | qnrC |
| D_T2 - D_T3 | -0.59 | 0.55 | qnrC |
| A_T1 - D_T4 | -0.69 | 0.49 | qnrC |
| A_T2 - D_T4 | -1.71 | 0.09 | qnrC |
| A_T3 - D_T4 | -1.37 | 0.17 | qnrC |
| A_T4 - D_T4 | -0.86 | 0.39 | qnrC |
| B_T2 - D_T4 | -1.37 | 0.17 | qnrC |
| B_T3 - D_T4 | 0.86 | 0.39 | qnrC |
| B_T4 - D_T4 | 0.00 | 1.00 | qnrC |
| C_T2 - D_T4 | 0.51 | 0.61 | qnrC |
| C_T3 - D_T4 | -0.53 | 0.60 | qnrC |
| C_T4 - D_T4 | -0.17 | 0.86 | qnrC |
| D_T2 - D_T4 | -0.51 | 0.61 | qnrC |
| D_T3 - D_T4 | 0.17 | 0.86 | qnrC |
| A_T1 - A_T2 | 0.89 | 0.37 | qnrS |
| A_T1 - A_T3 | 1.19 | 0.23 | qnrS |
| A_T2 - A_T3 | 0.30 | 0.77 | qnrS |
| A_T1 - A_T4 | 0.74 | 0.46 | qnrS |
| A_T2 - A_T4 | -0.15 | 0.88 | qnrS |
| A_T3 - A_T4 | -0.45 | 0.66 | qnrS |
| A_T1 - B_T2 | 0.43 | 0.67 | qnrS |
| A_T2 - B_T2 | -0.60 | 0.55 | qnrS |
| A_T3 - B_T2 | -0.94 | 0.35 | qnrS |
| A_T4 - B_T2 | -0.43 | 0.67 | qnrS |
| A_T1 - B_T3 | -0.45 | 0.66 | qnrS |
| A_T2 - B_T3 | -1.34 | 0.18 | qnrS |
| A_T3 - B_T3 | -1.63 | 0.10 | qnrS |
| A_T4 - B_T3 | -1.19 | 0.23 | qnrS |
| B_T2 - B_T3 | -0.94 | 0.35 | qnrS |
| A_T1 - B_T4 | -0.15 | 0.88 | qnrS |
| A_T2 - B_T4 | -1.04 | 0.30 | qnrS |
| A_T3 - B_T4 | -1.34 | 0.18 | qnrS |
| A_T4 - B_T4 | -0.89 | 0.37 | qnrS |
| B_T2 - B_T4 | -0.60 | 0.55 | qnrS |
| B_T3 - B_T4 | 0.30 | 0.77 | qnrS |
| A_T1 - C_T2 | 0.30 | 0.77 | qnrS |
| A_T2 - C_T2 | -0.59 | 0.55 | qnrS |
| A_T3 - C_T2 | -0.89 | 0.37 | qnrS |
| A_T4 - C_T2 | -0.45 | 0.66 | qnrS |
| B_T2 - C_T2 | -0.09 | 0.93 | qnrS |
| B_T3 - C_T2 | 0.74 | 0.46 | qnrS |
| B_T4 - C_T2 | 0.45 | 0.66 | qnrS |
| A_T1 - C_T3 | 0.17 | 0.86 | qnrS |
| A_T2 - C_T3 | -0.86 | 0.39 | qnrS |
| A_T3 - C_T3 | -1.20 | 0.23 | qnrS |
| A_T4 - C_T3 | -0.69 | 0.49 | qnrS |
| B_T2 - C_T3 | -0.32 | 0.75 | qnrS |
| B_T3 - C_T3 | 0.69 | 0.49 | qnrS |
| B_T4 - C_T3 | 0.34 | 0.73 | qnrS |
| C_T2 - C_T3 | -0.17 | 0.86 | qnrS |
| A_T1 - C_T4 | 0.45 | 0.66 | qnrS |
| A_T2 - C_T4 | -0.45 | 0.66 | qnrS |
| A_T3 - C_T4 | -0.74 | 0.46 | qnrS |
| A_T4 - C_T4 | -0.30 | 0.77 | qnrS |
| B_T2 - C_T4 | 0.09 | 0.93 | qnrS |
| B_T3 - C_T4 | 0.89 | 0.37 | qnrS |
| B_T4 - C_T4 | 0.59 | 0.55 | qnrS |
| C_T2 - C_T4 | 0.15 | 0.88 | qnrS |
| C_T3 - C_T4 | 0.34 | 0.73 | qnrS |
| A_T1 - D_T2 | -1.04 | 0.30 | qnrS |
| A_T2 - D_T2 | -1.93 | 0.05 | qnrS |
| A_T3 - D_T2 | -2.23 | 0.03 | qnrS |
| A_T4 - D_T2 | -1.78 | 0.07 | qnrS |
| B_T2 - D_T2 | -1.63 | 0.10 | qnrS |
| B_T3 - D_T2 | -0.59 | 0.55 | qnrS |
| B_T4 - D_T2 | -0.89 | 0.37 | qnrS |
| C_T2 - D_T2 | -1.34 | 0.18 | qnrS |
| C_T3 - D_T2 | -1.37 | 0.17 | qnrS |
| C_T4 - D_T2 | -1.49 | 0.14 | qnrS |
| A_T1 - D_T3 | -0.59 | 0.55 | qnrS |
| A_T2 - D_T3 | -1.49 | 0.14 | qnrS |
| A_T3 - D_T3 | -1.78 | 0.07 | qnrS |
| A_T4 - D_T3 | -1.34 | 0.18 | qnrS |
| B_T2 - D_T3 | -1.11 | 0.26 | qnrS |
| B_T3 - D_T3 | -0.15 | 0.88 | qnrS |
| B_T4 - D_T3 | -0.45 | 0.66 | qnrS |
| C_T2 - D_T3 | -0.89 | 0.37 | qnrS |
| C_T3 - D_T3 | -0.86 | 0.39 | qnrS |
| C_T4 - D_T3 | -1.04 | 0.30 | qnrS |
| D_T2 - D_T3 | 0.45 | 0.66 | qnrS |
| A_T1 - D_T4 | -0.69 | 0.49 | qnrS |
| A_T2 - D_T4 | -1.71 | 0.09 | qnrS |
| A_T3 - D_T4 | -2.06 | 0.04 | qnrS |
| A_T4 - D_T4 | -1.54 | 0.12 | qnrS |
| B_T2 - D_T4 | -1.37 | 0.17 | qnrS |
| B_T3 - D_T4 | -0.17 | 0.86 | qnrS |
| B_T4 - D_T4 | -0.51 | 0.61 | qnrS |
| C_T2 - D_T4 | -1.03 | 0.30 | qnrS |
| C_T3 - D_T4 | -1.05 | 0.29 | qnrS |
| C_T4 - D_T4 | -1.20 | 0.23 | qnrS |
| D_T2 - D_T4 | 0.51 | 0.61 | qnrS |
| D_T3 - D_T4 | 0.00 | 1.00 | qnrS |

**Supplementary Table S7**

Dunn’s post hoc comparisons of *qnr* genes abundances.

**References**

Enyoh, C. E., Wang, Q., Lu, S., 2023. Optimizing the efficient removal of ciprofloxacin from aqueous solutions by polyethylene terephthalate microplastics using multivariate statistical approach. Chemical Engineering Science, 278, 118917. https://doi.org/10.1016/j.ces.2023.118917

Hansen, C.M., 2007. Hansen Solubility Parameters: A User’s Handbook, Second edition. CRC press.

Lin, J., Yan, D., Fu, J., Chen, Y., & Ou, H. (2020). Ultraviolet-C and vacuum ultraviolet inducing surface degradation of microplastics. Water Research, 186, 116360. <https://doi.org/10.1016/j.watres.2020.116360>

Liu, G., Zhu, Z., Yang, Y., Sun, Y., Yu, F., & Ma, J. (2019). Sorption behavior and mechanism of hydrophilic organic chemicals to virgin and aged microplastics in freshwater and seawater. Environmental Pollution, 246, 26-33. <https://doi.org/10.1016/j.envpol.2018.11.100>

Silva, D. M., Paleco, R., Traini, D., Sencadas, V., 2018. Development of ciprofloxacin-loaded poly (vinyl alcohol) dry powder formulations for lung delivery. International journal of pharmaceutics, 547(1-2), 114-121. <https://doi.org/10.1016/j.ijpharm.2018.05.060>

Yilimulati, M., Wang, L., Ma, X., Yang, C., & Habibul, N. (2021). Adsorption of ciprofloxacin to functionalized nano-sized polystyrene plastic: Kinetics, thermochemistry and toxicity. Science of the Total Environment, 750, 142370. <https://doi.org/10.1016/j.scitotenv.2020.142370>

**Supplementary Figure S1**

(A) Macroscopic images of biofilm formation on microplastic surfaces in treatments C and D. (B) Epifluorescence microscopy images (DAPI staining, 1000× magnification) showing microbial colonization and biofilm structure on microplastic fragments from treatments C and D.

**Supplementary Figure S2**

Boxplots showing significant differences in functional pathway abundances across treatments, as identified by Tukey’s post-hoc test (ko01501= beta-Lactam resistance; ko01502 = vancomycin resistance; ko01523 = antifolate resistance; ko02025 = biofilm formation- *Pseudomonas aeruginosa*; ko05111 = biofilm formation – *Vibrio* spp.)
